# Integrative sleep management: from molecular pathways to conventional and herbal treatments

**DOI:** 10.1007/s00210-025-04183-y

**Published:** 2025-05-08

**Authors:** Yasmina M. Ebrahim, Mohamed A. Sadek, Miral O. Sabry, Rana M. Lotfy, Ahmed M. El-Dessouki, Dina Abou-Hussein, Riham A. El-Shiekh, Rana M. ElBishbishy

**Affiliations:** 1 Department of Pharmacology and Toxicology, Faculty of Pharmacy, Cairo University, Cairo, 11562 Egypt; 2 Department of Pharmacology and Toxicology, College of Medicine, University of Arkansas for Medical Sciences, Little Rock, AR 72205 USA; 3 Faculty of Science, National University of Singapore, Singapore Institute of Manufacturing Technology (SIMTech), Agency for Science, Technology and Research (A*STAR), Singapore, Singapore; 4 Pharmacology and Toxicology Department, Faculty of Pharmacy, Ahram Canadian University, 6th of October City, 12566 Giza Egypt; 5 Department of Pharmacognosy, Faculty of Pharmacy, Cairo University, Cairo, 11562 Egypt

**Keywords:** Insomnia, Nutraceuticals, Sleep disorders, Risk factors

## Abstract

Sleep is regarded as one of the most crucial factors in keeping a healthy lifestyle. To function normally, a person needs at least 6–8 h of sleep per day. Sleep influences not only our mood but also the efficiency with which we complete tasks. Sleep disorders exhibit diverse etiologies across different conditions and populations, with genetic and environmental factors playing a significant role in their development. Many issues emerge as a result of inadequate sleep. Unhealthy food and lifestyle choices have increased our susceptibility to sleep disorders. A well-balanced diet rich in essential vitamins and minerals can have a profound impact on sleep patterns, enhancing both the duration and quality of rest. The primary categories of sleep disorders include insomnia, sleep apnea (SA), narcolepsy, parasomnias, circadian rhythm disorders, and restless legs syndrome (RLS). The drugs used to treat sleep disorders are primarily habit-forming and have a history of withdrawal effects. This insufficiency in medication has prompted the hunt for newer, better options. Nutraceuticals are well-suited to the treatment of such illnesses. Its non-toxic, non-habit-forming properties, and practical efficiency have made it an outstanding choice. This review provides nutraceuticals used in sleep disorders. A comprehensive literature search was conducted utilizing several databases, including Google Scholar, Elsevier, Springer Nature, Wiley, PubMed, and EKB. Nutraceuticals are products that employ food or dietary components to treat or prevent disease. In the therapy of sleep disorders, nutraceuticals such as *Artemisia annua*, valerian, rosemary, jujube, Passionflower, lemon balm, ashwagandha, kava-kava, lavender, and chamomile have been shown to have remarkable benefits. These remedies exert their effects through multiple mechanisms, both directly by modulating neurotransmitter and hormonal pathways within sleep circuits, and indirectly by enhancing sleep quality through the alleviation of stress, inflammation, and oxidative stress. Clinical studies were piloted to validate the efficacy of natural sleep aids. Future research should focus on elucidating the precise mechanisms through which natural products influence sleep.

## Introduction

Sleep is an important part of human health since it affects cognitive function, emotional management, physical health, and quality of life (Buysse 2014). However, sleep disorders, particularly insomnia, have become more common in modern culture. About 50–70 million persons in the USA suffer from sleep disorders, with insomnia being the most frequent (Roth 2007a). Insomnia is characterized by difficulties getting asleep, sustaining sleep, or receiving restorative sleep, resulting in daily impairments such as exhaustion, mood disorders, and poor performance (Morin et al. 2015). Conventional treatments for insomnia frequently entail pharmacological treatment, such as benzodiazepines or benzodiazepine receptor agonists (Riemann et al. 2017). Although these drugs may be beneficial in the short-term, they are associated with a variety of side effects, including dependence, tolerance, and severe responses (Kripke 2018). Furthermore, long-term use of these drugs can result in rebound insomnia and withdrawal symptoms after termination (Lader 2011).

Herbal/natural products are one of the most popular forms of complementary and alternative medicine (CAM) (Ni et al. 2002). With the advent of self-managed health care, many people are turning to natural remedies (herbs, vitamins, and mineral supplements) as health promoting strategies or solutions to health problems (Chang 2000). They are widely available, can be purchased at supermarkets, pharmacies, and health food stores, and can be consumed without supervision, leading to the perception that these items are necessarily safe and free of health hazards (Ernst 2006). Some herbs and nutritional supplements that have been touted as sleep aids include Valerian root, St. John’s Wort, kava, passionflower, and melatonin (Ramakrishnan 2007). These supplements frequently include substances with sedative, anxiolytic, or sleep-promoting characteristics, like flavonoids, terpenes, and amino acids (Shi et al. 2014). The objective of this review was to trace the use of natural products as sleep aids with documentation of their roles in the management of sleep disorders. Additionally, this review encompassed a broad analysis of sleep disorders, including their types, causes, epidemiology, risk factors, pathogenesis, as well as both pharmacological and non-pharmacological therapeutic approaches.

## Search strategy

A search using Google Scholar, Elsevier, Springer Nature, Wiley, PubMed, and EKB was conducted. The following Mesh items were used “sleep disorders,” “Types of sleep disorders,” “natural products,” “causes,” “pathogenesis,” “symptoms,” “risk factors,” “epidemiology,” and “conventional treatments.”

## Types of sleep disorders

**Fig. 1:**
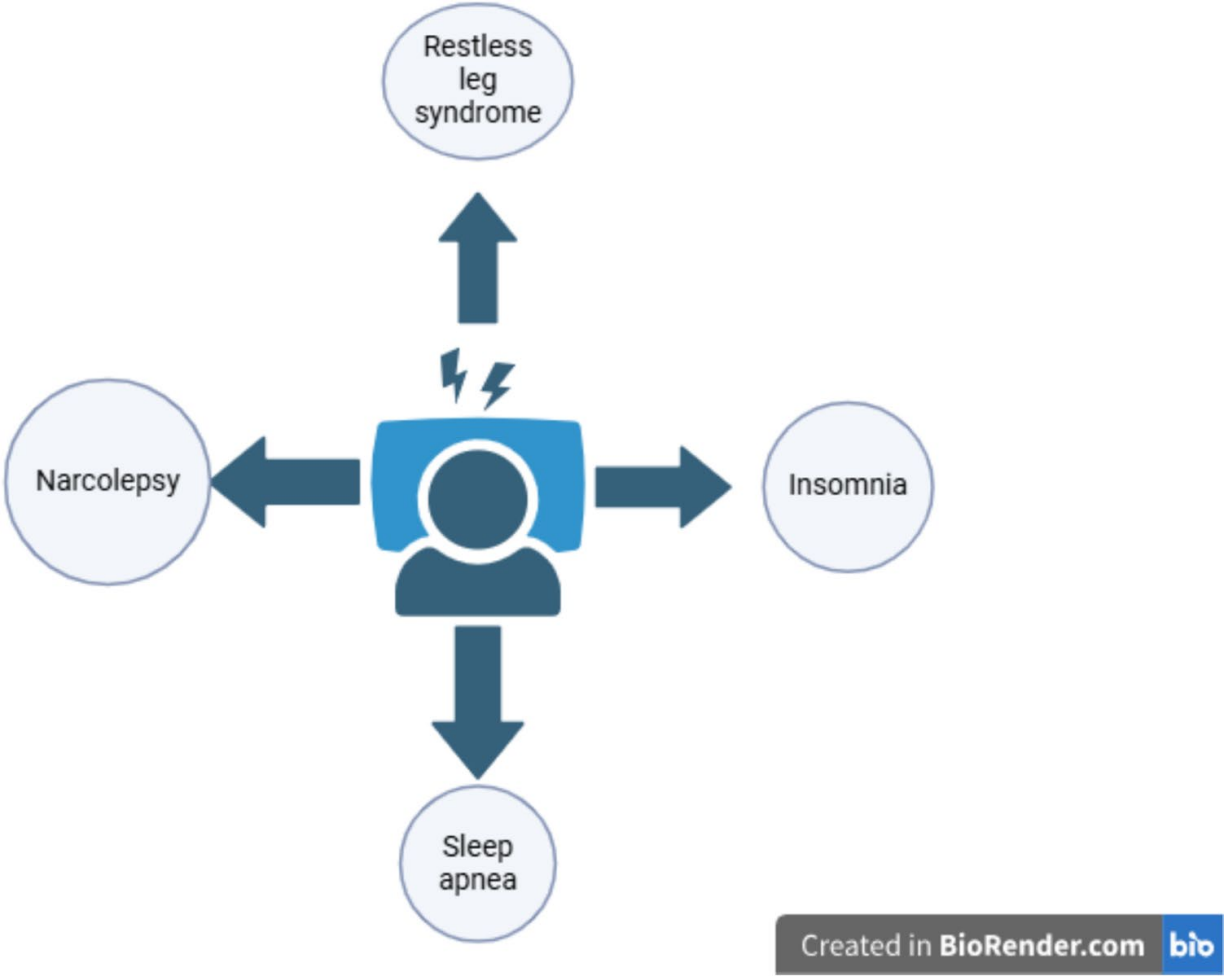
Types of sleep disorders. “Created with BioRender.com.”

Sleep disorders are a broad spectrum of conditions that can significantly affect health, safety, and quality of life. Insomnia, sleep apnea (SA), narcolepsy, parasomnias, circadian rhythm disorder, and restless leg syndrome (RLS) are some of the primary types of sleep disorders (Fig. 1).

### Insomnia

Insomnia disorder encompasses a plethora of symptoms during night and day that greatly influences wellbeing and quality of life. Some of the night complaints are prolonged onset of sleep, persistent difficulty in maintaining sleep and early morning wakefulness. Common daytimes problems are tiredness, compromised cognitive functions, anxiousness, diminished attention, and a depressed mood (Riemann et al. 2022). Insomnia can be classified into two subtypes: symptoms lasting for less than 3 months and are usually due to a sudden event or stress are classified as acute insomnia, while symptoms extending for more than 3 months is classified as chronic insomnia. Managing chronic insomnia is much harder due to distress evolving from the lack of sleep which can possibly deteriorate sleep over time (Strickland 2022). Zeng et al. concluded in a meta-analysis that insomnia is more prevalent in females than males (Zeng et al. 2020).

### Sleep apnea

A diminished airflow inspiration lasting for at least 10 is apnea while hypopnea is a lesser decline of airflow lasting 10 s or longer. Both apnea or hypopnea are categorized as obstructive or central (Javaheri et al. 2017).

**Table:** 

Central sleep apnea (CSA)	Obstructive sleep apnea (OSA)
Happens when a temporary decrease in generation of beathing rhythm	Happens when there is a complete blockage of the upper airway (tongue falling backward)

Snoring, choking sensation, difficulty in maintaining sleep, and non-restorative sleep are all symptoms that patients with OSA associate with. Obesity, family history of OSA, and small oropharyngeal airway are suggestive indicators for the disease. Gas exchange impairment causes hypercapnia, decreased oxygen saturation, and disrupted sleep, all lead to the repercussions of OSA, such as cardiovascular, metabolic, and neurocognitive effects. It has been noted that OSA is more prevalent in males than females (Jordan et al. 2014; Abbasi et al. 2021).

### Restless leg syndrome

RLS is a sensory motor disorder associated with sleep; its pathophysiology is yet to be clear. RLS is marked by a strong need to move that need is intensified with rest and alleviates with movement (Manconi et al. 2012).

Symptoms can range in frequency (occurring once per year to daily) and severity (from mild symptoms to severe effects on sleep and quality of life). Depression and suicide have been noted with very severe cases (Para et al. 2019). RLS is more prevalent in females than males with 30–50% higher occurrence in females. RLS was noted to be more prevalent in pregnant females with a chance of occurrence of one in five pregnant females (Manconi et al. 2012).

### Narcolepsy

Narcolepsy is a chronic disorder that usually commence at adolescence, and is mainly marked by excessive daytime sleepiness and, in a significant number of patients, cataplexy which is a sudden loss of muscle tone despite wakefulness that is triggered by strong emotion. Patients with narcolepsy often encounter many obstacles in maintaining employment, accessing education, reduced quality of life, and socioeconomic problems. Earlier, according to the presence or absence of cataplexy, narcolepsy was classified into two type types: narcolepsy type 1 and narcolepsy type 2 (Kornum et al. 2017).

In the 2014 edition of the International Classification of Sleep Disorders, narcolepsy has been redivided into narcolepsy type I (NT1) and narcolepsy type II (NT2), based on the absence or presence of orexins. Orexins are neuropeptides which contribute to the regulation of sleep and wakefulness. Orexins were considered a significant marker for cataplexy associated narcolepsy. Low level of orexins were in the cerebrospinal fluid of patient with NT1 and associated with cataplexy, on the other hand, NT2 had normal level of orexins and do not have cataplexy (Sateia 2014). It was noted that narcolepsy was more prevalent in men than women (1.6–1.8 males per 1 female) (Silber et al. 2002).

## Epidemiology

Sleep disorders are prevalent across different populations and commonly associated with various health conditions. In a cross-sectional study carried out by McArdle et al. on young adults, it was observed that, generally, at least one sleep disorder was found in 41% of females. Regarding the percentage in males, it was found to be 42.3% (McArdle et al. 2020). In specific, insomnia is the most common sleep disorder. Insomnia, which is characterized by difficulty in initiating sleep, continuing sleep, or poor sleep, was estimated to affect 13.9% of the population with a higher prevalence in females compared to males (Mai and Buysse 2008; Morin et al. 2020).

Another type of sleep disorder is the SA which is divided into OSA and CSA. In USA, the prevalence of mild OSA among adults aged 30 to 70 years was estimated as 14% for men and 5% for women, and the estimated prevalence of moderate to severe OSA was approximately 13% for males versus 6% for females (US Preventive Services Task Force et al. 2022). CSA is less prevalent compared to OSA (Ishikawa and Oks 2021). Additionally, CSA is rare in women compared to men, where an overall prevalence of CSA is 0.3% in females against 7.8% in males (Badr et al. 2019).

Regarding the prevalence of RLS in the general population, it is postulated to be around 10% (Phillips et al. 2000). However, this percentage can vary considerably in different age groups. In younger adults with an age range from 18 to 29, the prevalence is approximately 3%, while in geriatric population (> 80 years), the percentage is around 19% (Phillips et al. 2000).

On the other hand, narcolepsy, which is characterized by excessive day-time sleepiness, is considered a rare sleep disorder (Chavda et al. 2022; Manfredi et al. 1987). Narcolepsy has two types depending on the presence or the absence of cataplexy. NT1, which is associated with cataplexy, has a prevalence of 12.6/100,000 individuals, whereas NT2, that lacks cataplexy, has a prevalence of 25.1 per 100,000 individuals (Ohayon et al. 2023).

Interestingly, sleep disorders are usually comorbid with other medical conditions. As an example, patients suffering from episodic migraine have a higher risk of insomnia and RLS (Vgontzas et al. 2023). Likewise, patients with epilepsy exhibited higher prevalence of RLS (20.6% against 6.1% in controls) (Khachatryan et al. 2020). Moreover, sleep disorders are commonly encountered in various neurological disorders, including amyotrophic lateral sclerosis, multiple system atrophy, and Parkinson’s disease (Taximaimaiti et al. 2021; Anghel et al. 2023). Furthermore, sleep disorders are usually observed in patients having chronic obstructive pulmonary disease (COPD) and cardiovascular diseases (Vanfleteren et al. 2020; Wang et al. 2021).

## Pathogenesis

Sleep disorders have various etiologies across different conditions and populations. Indeed, genetics and environmental conditions play a significant role in most sleep disorders (Palagini et al. 2023; Bidaki et al. 2012). Regarding insomnia, there is an interplay between biochemical, neuroendocrine, immune, and psychosocial factors (Kang et al. 2022). On the biochemical level, it was observed that people with insomnia have a higher metabolic rate compared to normal individuals. This was assessed via measuring the oxygen consumption (Bonnet and Arand 1998). Additionally, the neuroendocrine causes of hyperarousal were highlighted in a previous study. Interestingly, patients suffering from insomnia exhibited an activation in the stress response system. This claim was proved by measuring urinary cortisol concentrations, where people with insomnia showed a higher level of urinary cortisol compared to normal subjects. This finding confirms the involvement of the hypothalamic–pituitary–adrenal (HPA) axis in the pathology of insomnia (Vgontzas et al. 2001; Vgontzas et al. 1997). Furthermore, immunity dysfunction was related to insomnia. A greater ratio between the inflammatory cytokines to the anti-inflammatory cytokines was witnessed in insomnia patients (Akkaoui et al. 2023). Moreover, psychological stress, indeed, leads to a hyperarousal state (Harvey 2002).

Concerning the pathogenesis of SA, OSA is mainly due to frequent collapse in the upper airway leading to hypercapnia, hypoxia, and sleeping disturbances (Lv et al. 2023). This collapse is caused by several reasons, like obesity, alteration in the upper airway function, or pharyngeal neuropathy (Lv et al. 2023). In fact, adenoid and/or tonsil hypertrophy are the most common causes of OSA in children, while obesity is the most prevalent cause in older children and adolescents (Mussi et al. 2023). On the other hand, CSA is an abnormal ventilatory drive, causing interruption or reduction in breathing without effort during sleep (Ishikawa and Oks 2021). Breathing interruption may be idiopathic, or due to heart failure, high altitudes, drug-induced, brainstem and spinal cord lesions, congenital, or because of neuromuscular or skeletal disorders (Hernandez and Patil 2016).

Furthermore, the pathogenesis of RLS mainly involves a neural contribution. It is hypothesized that dopamine (DA) deficiency is accused for the RLS. This postulation arose since the dopaminergic drugs and dopamine agonists were found to be effective in treating the condition (Lv et al. 2021). Noteworthy, unlike Parkinson’s disease, DA deficiency in RLS is not in the substantia nigra (Lv et al. 2021). Fascinatingly, brain iron deficiency is linked to dopaminergic abnormality (Nanayakkara et al. 2023). In addition, electrophysiological findings indicate the interplay of various generators, causing an increase in the nervous system excitability and alterations in inhibition within somatosensory and nociceptive pathways (Antelmi et al. 2024).

Regarding the pathogenesis of narcolepsy, NT1 is a result of losing hypocretin (orexin)-producing neurons in the hypothalamus, causing a disruption in the sleep–wake cycles (Liblau et al. 2024; Franceschini et al. 2021). Hypocretin is a wake-promoting peptide. The loss of the hypocretin-producing neurons is intensely associated linked to an autoimmune process, where NT1 is correlated to MHC class II allele, HLA-DQB1 * 06:02, and T cell receptor genes (Liblau et al. 2024; Ollila et al. 2023). Furthermore, heightened T cell reactivity against hypocretin has been described in NT1 patients (Kornum 2020). Interestingly, corticotropin-releasing hormone (CRH)-positive neurons in the paraventricular nucleus of NT1 post-mortem brains have witnessed a considerable decline by 88%; however, other hypothalamic cell groups remained unaffected (Shan et al. 2022). Moreover, H1 N1 influenza A infection and immunization with Pandemrix® were identified as environmental risk factors triggering NT1 development (Ollila et al. 2023). On the other hand, NT2 pathogenesis is not yet fully established (Miyagawa and Tokunaga 2019).

## Causes

**Fig. 2:**
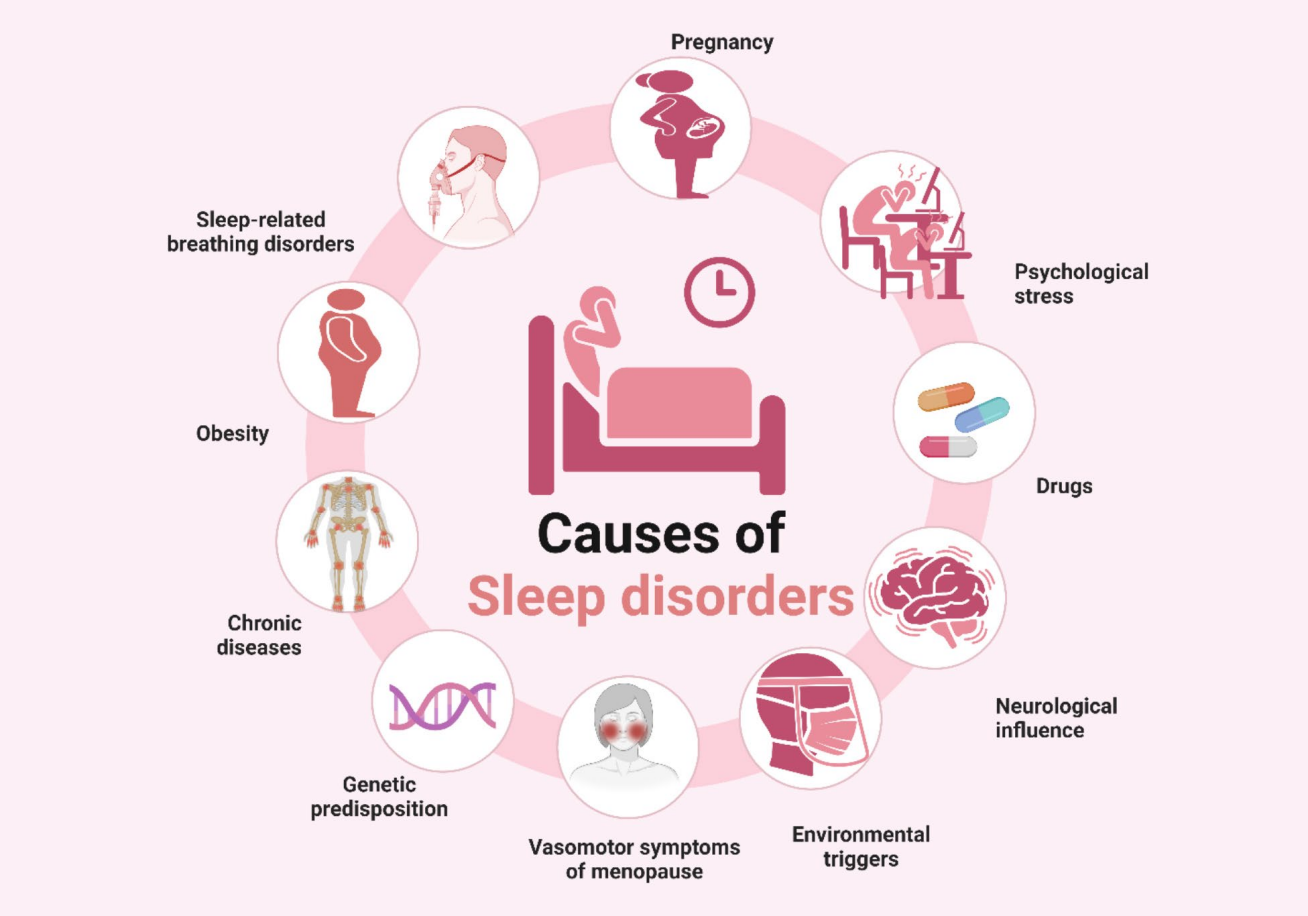
Causes of sleep disorders. “Created with BioRender.com”

Sleep disorders are multifactorial (Fig. 2). It can be caused by genetic factors, environmental triggers, or neurological influences (Caylak 2009). Sleep disorders could be an outcome of a certain medical condition. For example, vasomotor symptoms associated with menopause can cause sleep disturbances (Lee et al. 2019). In addition, chronic diseases such as asthma, chronic obstructive pulmonary disease, and rheumatoid arthritis can impose a negative influence on the quality of sleep (Smolensky et al. 2011). Furthermore, obesity, and some drugs can cause respiratory disturbances, thus, increasing the possibility of sleep-related breathing disorders (Romero-Corral et al. 2010). Moreover, leg cramps and RLS were commonly reported in pregnant women (Hensley 2009). Finally, daily life stressors execute a psychological pressure which can indeed have a negative impact on sleep quality, depth, and efficiency (Yoo et al. 2023).

## Symptoms

**Fig. 3:**
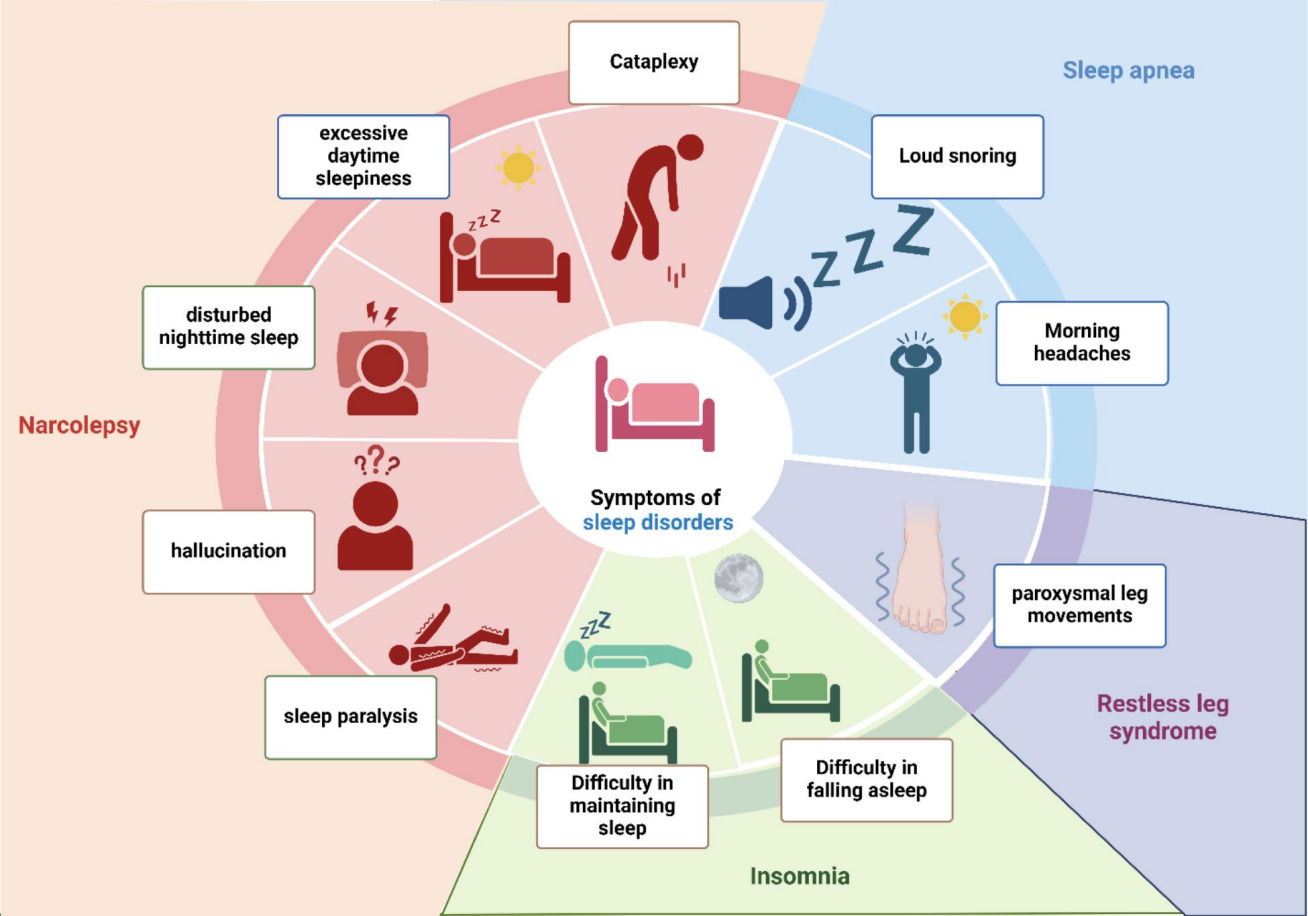
Symptoms of sleep disorders. “Created with BioRender.com”

Symptoms of sleep disorders vary according to the type of disorder (Fig. 3). Patients suffering from insomnia experience symptoms of difficulty in falling asleep and/or maintaining sleep (Roth 2007b). In SA, patients may show signs of loud snoring, dry mouth upon waking up, and morning headaches (Spalka et al. 2020). Regarding patients with RLS, they report the symptoms as electric current along their legs leading to a paroxysmal leg movement (Byrne et al. 2006). On the other hand, symptoms of narcolepsy revolve around four main symptoms: excessive daytime sleepiness, sleep paralysis, disturbed nighttime sleep, hallucinations, with or without cataplexy (Chavda et al. 2022). Noteworthy, NT1 is the type of narcolepsy associated with cataplexy while NT2 is not accompanied by cataplexy (Ohayon et al. 2023).

## Risk factors

Risk factors contributing to sleep disorders are numerous. According to prospective study, gender is a risk factor, where females are more prone to sleep disturbances than males; however, the exact reason is not known (Smagula et al. 2016). Additionally, depressed mood promotes sleep disorders. Fairly, the relation between depression and sleeplessness is bidirectional, since sleep disturbances results in a depressed mode as well (Smagula et al. 2016). Moreover, physical illness (like hypertension and dyslipidemia) and psychiatric comorbidities (like anxiety) are considered robust risk factors for sleep disorders (Hwang et al. 2022). In addition, chronic stress was found to alter the melatonin-related pathways in animal models, thus triggering sleep disorders (Xia et al. 2023). Furthermore, obesity is considered a potent risk factor for SA (Brzecka et al. 2020). Noteworthy, aging solely is not considered a predictor for sleep disorder; however, it increases the risk of development of other risk factors (Smagula et al. 2016).

## Impact on health

Indeed, any disorder affecting sleep quality will have a negative impact on both physical and mental well-being. Consequences on adolescents are critical as sleep disorders affect their overall health, behavior, mood, and academic performance during a crucial stage of their physical and emotional development (Kansagra 2020). In adults, the outcomes of sleep disorders are related to brain health. Patients exhibited a wide range of CNS disorders ranging from stroke to subclinical cerebrovascular disease and cognitive decline, including the development of Alzheimer’s disease and related dementias (Gottesman et al. 2024).

Moreover, in cancer patients, sleep disorders are widely prevalent, affecting 30–50% of patients. Unfortunately, these disorders can drastically impact on the patients’ quality of life as it is linked to the pain, anxiety, and depression associated with the disease (Strik et al. 2021). Furthermore, there is a substantial bidirectional relationship between cardiovascular diseases (CVDs) and sleep disorders. It is proven that inflammation, sympathetic activation, and endothelial dysfunction play serious roles in sleep disorders, all of which are predisposing factors for CVDs (Wang et al. 2021). Additionally, the influence of sleep disorders extends to pregnancy. Commonly, pregnant women suffer from RLS leading to a negative impact on the sleep quality of pregnant women (Gupta et al. 2016). This can affect maternal and fetal health, thus, possibly contributing to conditions such as preeclampsia and gestational diabetes (Kember et al. 2023).

## Sleep as a complex physiological process: neurobiology and molecular physiology

### Sleep cycle and endocrine interactions

Sleep is a multifaceted natural phenomenon in most animal kingdoms (Andrillon and Oudiette 2023). It is generally characterized by unconsciousness and reduced responsiveness in which individuals preserve and replenish their energy, maintaining normal physiological and mental activities (Eugene and Masiak 2015). However, it could be easily distinguished from other unconsciousness conditions (coma, seizures, or anesthesia) because sleep is habitually reversed to wakefulness (Joiner 2018). This sleep–wake cycle is maintained by a complex interplay of circadian and homeostatic processes involving various neural circuits and molecular mechanisms, and its disturbance provokes sleep disorders (Eban-Rothschild et al. 2018). Noteworthy, sleep and endocrine system are closely connected as they both regulate each other (Smith and Mong 2019). This section will provide insights into the sleep cycle, sleep-endocrine axis, and the molecular physiology of sleep that could be therapeutically manipulated to manage various sleep disorders.

Brain electrical activity is waving during sleep, classifying sleep into the rapid eye movement (REM) stage and non-REM (NREM) stage (Vyazovskiy et al. 2011). Further investigation classified NREM into three sub-stages in which brain activities and sleep deepness are dissimilar (Patel et al. 2024). Surprisingly, Patel et al. underscored that sleep disorders occur in different sleep cycles; subsequently, diagnosis and intervention will vary among them (Patel et al. 2024). On one hand, REM sleep disorder, insomnia, and narcolepsy could be coupled to the REM phase (Bramich et al. 2023; Feige et al. 2018; Thorpy et al. 2024). On the other hand, somnambulism, sleep terrors, and sleep-related eating disorders are considered NREM parasomnias (Castelnovo et al. 2018). Moreover, SA could be linked to both REM and NREM phases (Alzoubaidi and Mokhlesi 2016).

**Fig. 4:**
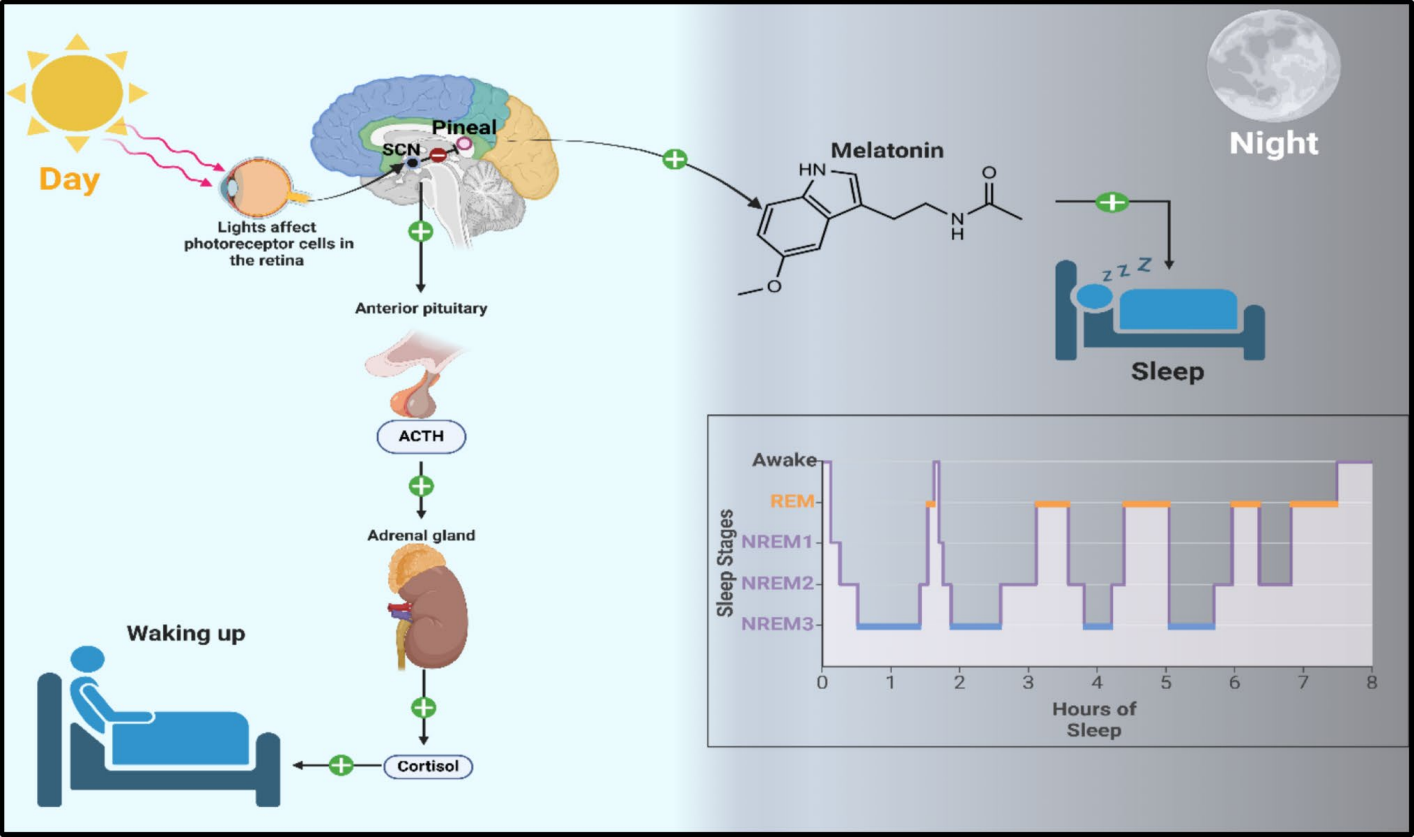
Illustration of retinohypothalamic tract and sleep stages “Created with BioRender.com.” SCN: Suprachiasmatic nucleus; REM: rapid eye movement; NREM: non-rapid eye movement

The suprachiasmatic nucleus (SCN), located in the hypothalamus, receives photic signals from the photoreceptors located in the retina, allowing the SCN to regulate the biological day and night cycle and behave as an internal clock (Welsh et al. 2010). Sleep and hormonal secretion are tightly interconnected; hormonal dysregulation provokes sleep disorders, and the latter could also aggravate hormonal abnormalities (Morgan and Tsai 2015). For instance, melatonin, the central sleeping hormone released at night, is controlled by SCN. Melatonin enhances sleep quality by acting on specific receptors to provoke sedation by suppressing SCN firing (Doghramji 2007). Moreover, melatonin also reduces the levels of the awakening hormone cortisol (Castano et al. 2019). Subsequently, insomnia may lead to stress and metabolic disturbance by upregulating cortisol (Hirotsu et al. 2015). Additionally, growth hormone amends wakefulness and is secreted mainly during deep sleep (Cauter and Plat 1996). Furthermore, prolactin and female gonadal hormone secretion increase during sleep (Liu and Park 1988). Figure 4 illustrates the retinohypothalamic tract that regulates sleep besides sleep stages.

### Sleep–wake cycle receptors

Melatonin, regulated by the SCN, is considered the master regulator of sleep that links internal physiological activities to the surrounding environmental changes (Doghramji 2007). Concisely, melatonin binds to three active sites, where the first two (MT_1_ and MT_2_) are considered G_i_-protein coupled receptors localized in the SCN, while MT_3_ is a reductase enzyme (Dubocovich 2007). Gobbi et al. reported that MT_1_ is involved mainly in REM regulation, while MT_2_ regulates NREM sleep (Gobbi and Comai 2019). In both cases, melatonin improves sleep quality by controlling the circadian rhythm and reducing body temperature, which increases sleepiness (Costello et al. 2014). Besides sleep adjustment, melatonin has various properties, including metabolic regulation, hormonal regulation, neuroprotection, antioxidant, and anti-aging (Arendt and Aulinas 2000). Remarkably, melatonin is biosynthesized from serotonin (Zhao et al. 2019), another sleep modulator (Portas et al. 2000). Serotonin’s impact on sleep is complex and controversial as it can stimulate sleep and alertness, depending on the site of action and the receptor type (Monti and Jantos 2008).

Gamma-aminobutyric acid (GABA) is the primary inhibitory neurotransmitter in mammals, located in several brain regions (Smart and Stephenson 2019). It acts by activating an ion-linked GABA_A_ receptor, promoting a rapid chloride influx, and inhibiting waking neurons (Allen et al. 2024). Interestingly, most drugs acting on GABA_A_ receptors are allosteric modulators, meaning they enhance the sedative influence of GABA but do not act by themselves (Wisden et al. 2019). GABA also binds to a G_i/o_ protein-coupled receptor, known as GABA_B_ receptor, that regulates potassium flux and induces a slow synaptic inhibition (Padgett and Slesinger 2010). Besides GABA_A_ and GABA_B_ receptors, GABA_C_ provides a sustained chloride influx (Gottesmann 2002). Interestingly, GABA is biosynthesized from glutamate, a negative sleep regulator (Kaczmarski et al. 2023).

Adenosine is usually produced from the metabolism of adenosine triphosphate, a crucial energy molecule, and exerts a plethora of physiological functions, including sleep regulation (Sheth et al. 2014). Adenosine’s influence on sleep is regulated, at least in part, by A_1_ and A_2 A_ receptors (Bjorness and Greene 2009). Concisely, the A_1_ receptor is a G_i_-protein coupled receptor, and its central activation amends arousing induced by cholinergic in the basal forebrain region, promoting sleep (Thakkar et al. 2003). Conversely, A_2 A_ receptors are G_s_ in nature, and their activation stimulates sleep neurons and modulates histaminergic and cholinergic neurons (Bjorness and Greene 2009). These outcomes are supported by the wakefulness induced by the pharmacological antagonism of adenosine receptors by caffeine or by genetic modification in adenosine receptor knock-out genotypes (Reichert et al. 2022).

**Fig. 5:**
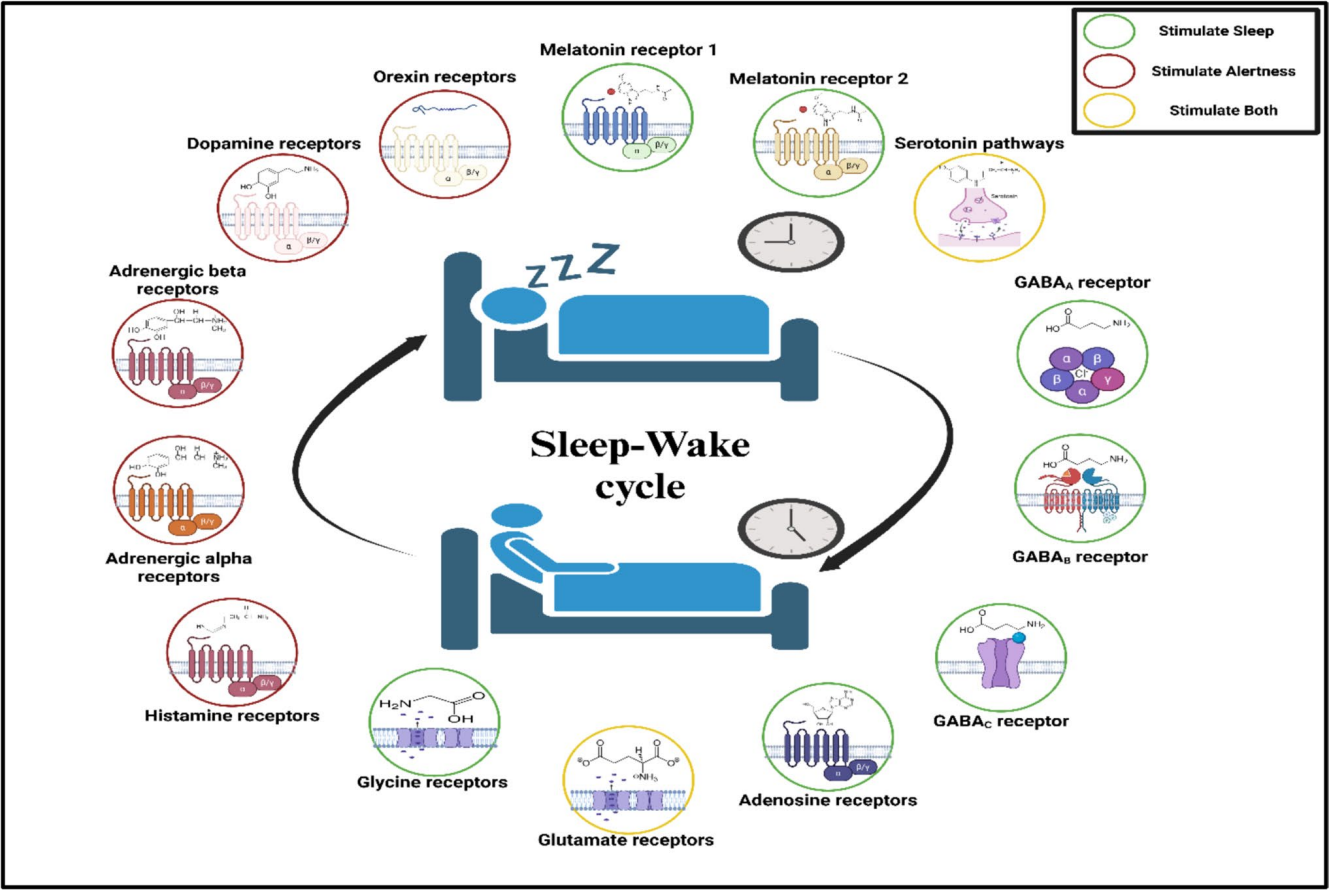
Influence of specific receptors involved in the sleep–wake cycle with a sketch of its ligand “Created with BioRender.com.” GABA: gamma-aminobutyric acid

A series of wakefulness receptors also control the sleep–wake cycle. For instance, as previously mentioned, glutamate is a significant wakefulness neurotransmitter (Kaczmarski et al. 2023). Glutamate binds to a plethora of receptors that provoke wakefulness, including AMPA (Yin et al. 2019), NMDA (Manfridi et al. 1999), and kainate receptors (Yin et al. 2019). Moreover, glutamate can regulate sleep and wakefulness (Shi and Yu 2013). Conversely, NMDA receptor activation by glycine can promote sleep (Kawai et al. 2015). Besides glutamate, histamine’s activation of the central H1 receptor promotes wakefulness, explaining the sedative effect of the H1 antagonist (Thakkar 2011). Noteworthy, H3 receptors are presynaptic receptors that negatively regulate histamine release, pointing to H3 agonists as a promising vigilance drug (Parmentier et al. 2007). Moreover, noradrenaline and its precursor, dopamine, control alertness through their central receptors (Ranjbar-Slamloo and Fazlali 2019). To elaborate, noradrenaline released in locus coeruleus enhances arousal via alpha and beta receptors (Berridge et al. 2012). Furthermore, D1 and D2 receptors activated by dopamine inhibit sleepiness (Zhang et al. 2022). Additionally, orexin (OX) receptors (OX1 and OX2) are crucial for arousal, underscoring the possible loss of OX-generating neurons as a pathophysiological event in narcolepsy (Scammell and Winrow 2011). Figure 5 summarizes receptors involved in the sleep–wake cycle.

The chemical structure of all ligands is drawn except orexin since it is a protein in nature with a complex chemical structure; hence, an amino acid chain is used to present orexin.

## Therapy of sleep disorders

### Non-drug therapies and lifestyle changes in the management of sleep disorders

Sleep disorders, including insomnia, OSA, and RLS, are widespread health concerns that significantly impact individuals’ quality of life (Kim, et al. 2024). These conditions are linked to various comorbidities, such as cardiovascular disease, obesity, and mental health disorders (Laaboub et al. 2022; Duraccio et al. 2022; Palagini et al. 2022). While pharmacological treatments are commonly used, there is growing interest in non-drug therapies and lifestyle modifications. The current findings examine the effectiveness of these interventions, focusing on behavioral therapies, sleep hygiene, physical activity, dietary changes, and other complementary approaches (Briguglio et al. 2020; Wilson et al. 2022, 2023).

### Cognitive Behavioral Therapy for Insomnia (CBT-I)

Cognitive Behavioral Therapy for Insomnia (CBT-I) is a first-line, non-pharmacological treatment for chronic insomnia. This structured program targets negative thoughts and behaviors contributing to sleep disturbances (Soong et al. 2021). Key techniques include as follows:

#### Cognitive restructuring

Identifying and correcting irrational thoughts related to sleep, such as excessive fear of sleeplessness or overestimating the impact of poor sleep. Patients learn to replace negative thoughts with rational perspectives that reduce stress and anxiety surrounding sleep (Redeker et al. 2020; Sweetman et al. 2021).

#### Stimulus control

Strengthening the association between bed and sleep by limiting bedroom activities to sleep and intimacy. This method discourages behaviors like working, eating, or watching television in bed, ensuring that the mind associates the bed with restful sleep (Iao et al. 2022; Jansson-Fröjmark et al. 2024).

#### Sleep restriction

Enhancing sleep efficiency by initially restricting time in bed to the actual sleep duration, then gradually increasing it. This process helps patients consolidate sleep and improve their ability to fall and stay asleep over time (Maurer et al. 2020; Maurer et al. 2021).

#### Relaxation training

Utilizing methods such as progressive muscle relaxation, deep breathing, and meditation to decrease physiological arousal before bedtime. These practices help to counteract stress-induced insomnia and create a calm pre-sleep routine (Liu et al. 2020; Toussaint et al. 2021).

Research has consistently demonstrated that CBT-I leads to sustained improvements in sleep quality and duration, often proving more effective and with fewer side effects than pharmacological treatments. The adaptability of CBT-I across diverse patient populations enhances its widespread acceptance and efficacy. Digital platforms and mobile applications now offer accessible CBT-I programs, broadening its reach and making effective sleep management more attainable for the general population (Climent-Sanz et al. 2022; Lu et al. 2023; Ntikoudi et al. 2024).

### Sleep hygiene education

Sleep hygiene encompasses behavioral and environmental practices that optimize sleep. These include as follows:

#### Regular sleep schedule

Maintaining consistent sleep and wake times, including weekends, to regulate circadian rhythms and reinforce the body’s natural sleep–wake cycle. Irregular sleep patterns disrupt biological clocks, making it harder to maintain consistent sleep quality (Walker et al. 2020; Basit et al. 2025).

#### Optimal sleep environment

Creating a quiet, dark, and cool bedroom with blackout curtains, white noise machines, or earplugs to minimize disturbances. Research suggests that cooler room temperatures, typically between 60 and 67 °F (16–19 °C), facilitate deeper sleep (Strøm-Tejsen et al. 2016; Raj et al. 1214).

#### Limiting stimulants

Avoiding caffeine, nicotine, and heavy meals near bedtime to prevent disruptions in the sleep cycle. Late-night consumption of alcohol, although initially sedating, can lead to fragmented sleep and frequent nighttime awakenings (Spadola et al. 2019).

#### Avoiding screen exposure before bed

Reducing exposure to blue light from electronic devices (phones, tablets, and computers) at least 1 h before sleep to support natural melatonin production. Blue light suppresses melatonin release, making it harder for the body to transition into sleep mode (Tähkämö et al. 2019; Shechter et al. 2020).

**Fig. 6:**
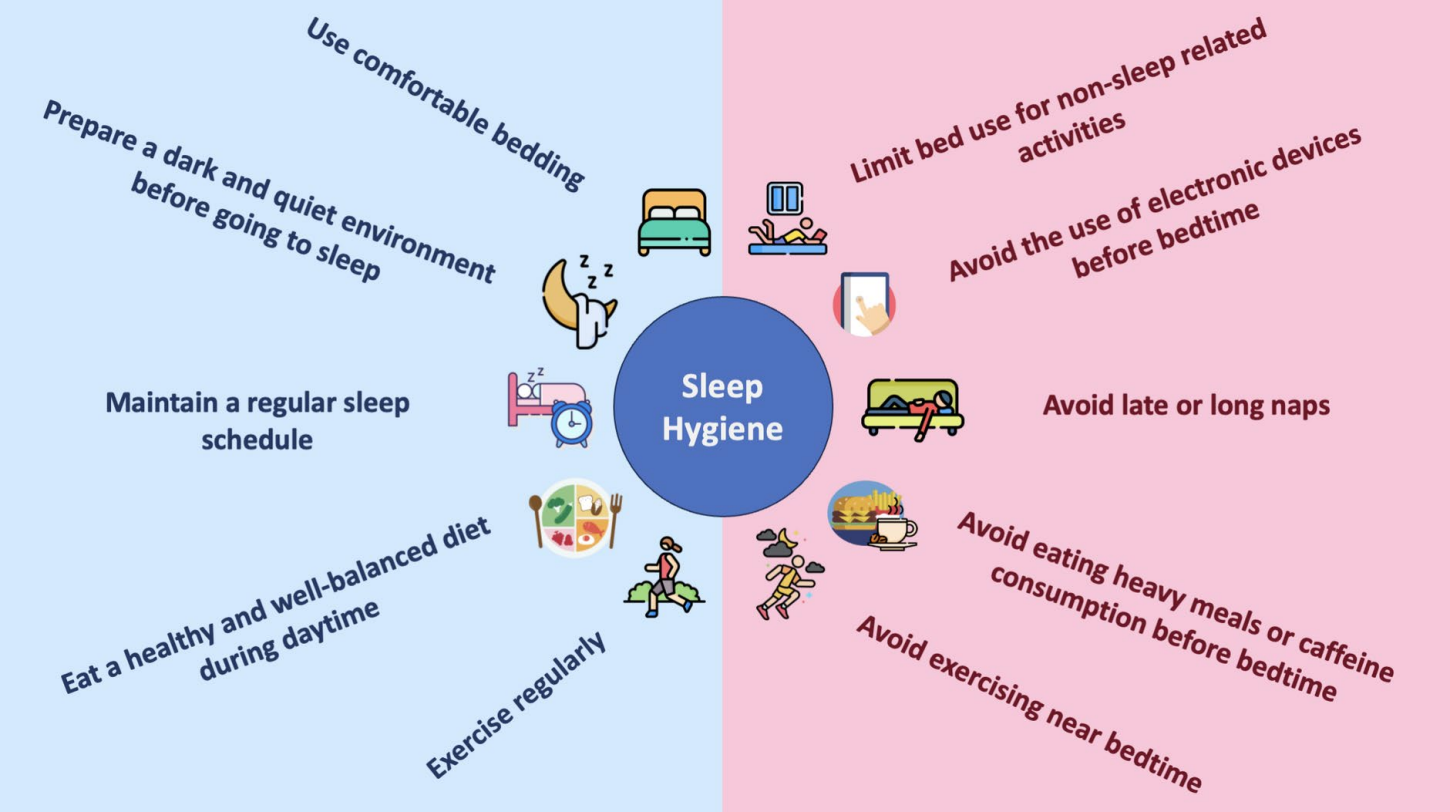
Sleep hygiene tips (icons by Falticon.com)

Educational programs emphasizing sleep hygiene have been shown to improve sleep quality and duration, especially when integrated with other behavioral therapies. Schools, workplaces, and public health campaigns are increasingly recognizing the importance of sleep education, promoting better sleep habits across all age groups (Redeker et al. 2019; Gaskin et al. 2024). The literature is packed with myriad evidence supporting the impact of simple practices (summarized in Fig. 6) on the quality and quantity of sleep especially in individuals with or prone to having sleep disorders (Varadharasu and Das 2024; Carrión-Pantoja et al. 2022; Corrêa et al. 2024; Norouzi et al. 2024).

### Physical activity and exercise

Engaging in physical activity contributes to improved sleep duration and quality through several mechanisms:

#### Endorphin release

Exercise triggers the release of endorphins, enhancing mood and reducing stress, thereby facilitating better sleep initiation. Additionally, physical activity increases body temperature, and the subsequent post-exercise temperature drop may aid in sleep induction (Basso and Suzuki 2017; Sepdanius et al. 2023).

#### Circadian rhythm regulation

Regular physical activity helps synchronize the body’s internal clock, promoting more consistent sleep and wake times. This is particularly beneficial for individuals suffering from circadian rhythm sleep disorders, such as delayed sleep phase disorder (Thomas 2020; Weinert and Gubin 2022).

#### Reduction in anxiety and depression symptoms

Since anxiety and depression often contribute to sleep disturbances, exercise serves as a natural remedy to alleviate these symptoms. Physical activity has been linked to increased production of neurotransmitters like serotonin and dopamine, which help regulate mood and improve sleep (Alnawwar et al. 2023).

#### Muscle relaxation and physical fatigue

Engaging in exercise can lead to muscle relaxation and physical fatigue, making it easier to fall asleep. However, the timing of exercise is crucial. While moderate morning and afternoon exercise has positive effects on sleep, high-intensity workouts close to bedtime can increase alertness and hinder the ability to fall asleep (Alkhaldi et al. 2023).

Studies suggest that moderate aerobic exercises such as walking, swimming, or cycling for at least 30 min several times a week yield the most significant sleep benefits. Resistance training and yoga have also been found to be beneficial, particularly in reducing stress-related insomnia (Wang and Boros 2021; Zhou et al. 2022).

### Dietary modifications

Diet plays a crucial role in sleep regulation, with specific nutrients influencing sleep quality by affecting neurotransmitters and circadian rhythms. A well-balanced diet that includes essential vitamins and minerals can significantly impact sleep patterns, improving both the duration and quality of rest (Alruwaili et al. 2023). Dietary interventions include as follows:

#### Melatonin-rich foods

Melatonin, a hormone that governs sleep–wake cycles, can be naturally supported by consuming foods rich in melatonin, such as tart cherries, grapes, bananas, nuts, and oats. These foods help promote a natural feeling of sleepiness and can assist in maintaining a steady sleep schedule (Pereira et al. 2020).

#### Magnesium and calcium

These essential minerals contribute to neurotransmitter regulation, promoting relaxation and calmness, which may aid in better sleep. Magnesium plays a role in reducing cortisol levels, a stress hormone that can disrupt sleep. Foods such as leafy greens, almonds, dairy products, seeds, and whole grains are excellent sources (Zhang et al. 2022; Briskey et al. 2024).

#### High glycemic foods

Diets high in refined carbohydrates and sugar can negatively impact sleep quality by causing fluctuations in blood sugar levels and subsequent insulin spikes. These sudden changes can lead to nighttime awakenings and difficulty maintaining a restful sleep. Opting for complex carbohydrates like whole grains, legumes, and fiber-rich vegetables can help stabilize blood sugar levels and support more consistent sleep patterns (Gangwisch et al. 2020).

Nutritional interventions in these areas have demonstrated promising results in enhancing sleep duration and quality, particularly when combined with other lifestyle modifications. Additionally, staying well hydrated and maintaining a consistent eating schedule can further help regulate sleep cycles (Hepsomali and Groeger 2021; Kesztyüs et al. 2021; Arab et al. 2023).

### Mindfulness and relaxation techniques

Mindfulness meditation, yoga, and relaxation techniques have emerged as effective complementary strategies for managing sleep disorders. These practices help reduce physiological and psychological stress, which can contribute to sleep disturbances. By promoting relaxation, these methods encourage the body’s natural transition into restful sleep (Wang et al. 2019; Rusch et al. 2019).

#### Mindfulness meditation

Engaging in mindfulness techniques that focus on present-moment awareness and acceptance can decrease sleep latency and enhance sleep quality, particularly for individuals with insomnia and anxiety-related sleep issues. Practicing mindfulness before bed can reduce the racing thoughts and mental clutter that often interfere with sleep (Wang et al. 2019).

#### Yoga

Gentle forms of yoga that incorporate breathing exercises and relaxation techniques have been shown to improve sleep quality, especially in individuals suffering from chronic insomnia or high stress levels. Poses such as child’s pose, legs-up-the-wall pose, and forward bends help calm the nervous system and prepare the body for rest (Turmel et al. 2022).

#### Progressive muscle relaxation (PMR)

PMR, a technique involving the tensing and relaxing of muscle groups, has proven effective in reducing sleep onset latency and extending sleep duration in individuals with insomnia. This practice helps release physical tension that may be preventing relaxation (Simon et al. 2022).

#### Breathing exercises

Controlled breathing techniques, such as the 4–7–8 method or diaphragmatic breathing, can slow the heart rate and activate the parasympathetic nervous system, signaling the body that it is time to sleep. Practicing these exercises regularly can help establish a bedtime routine that encourages relaxation (Jerath et al. 2019; Laborde et al. 2019).

These relaxation-focused methods foster a state of tranquility, making it easier to fall asleep and maintain restful sleep throughout the night. Integrating them into a nightly routine can enhance the effectiveness of other sleep-promoting strategies (Rusch et al. 2019).

### Pharmacological therapy

The list of pharmacological therapy of common sleep disorders is displayed in Table 1.

**Table 1:** Summary of the pharmacological therapy of insomnia, sleep apnea, restless leg syndrome, and narcolepsy

Sleep disorder	Pharmacological class	Examples	Guidelines and references
Insomnia	Benzodiazepines	Temazepam, triazolam	Riemann et al. 2023; Madari et al. 2021; Sateia et al. 2017
	Benzodiazepine receptor agonists	Eszopiclone, zopiclone, zolpidem, zaleplone	
	Orexin receptor antagonist	Suvorexant, daridorexant	
	Sedative norepinephrine/serotonin enhancers	◦ Tricyclic antidepressants: Doxepin ◦ Serotonin receptor antagonists and reuptake inhibitors (SARI): Trazodone	
	Melatonin and melatonin receptor agonists	Melatonin, ramelteon	
	Histamine receptor antagonist	Diphenhydramine	
	Dopamine (D2)/serotonin (5HT2 A) receptors antagonists	Olanzapine, quetiapine	
Sleep apnea	Carbonic anhydrase inhibitors	Acetazolamide	Randerath et al. 2022; Nobre 2024; Arredondo, et al. 2022; Liu et al. 2024
	Selective norepinephrine reuptake inhibitor/muscarinic receptor antagonist combination therapy	Atomoxetine/oxybutynin combination therapy	
	Selective serotonin reuptake inhibitors	fluoxetine	
	Norepinephrine and dopamine reuptake inhibitors	Solriamfetol	
	Dopamine reuptake inhibitors	Modafinil, armodafinil	
	Glucagon-like peptide- 1 agonists	Tirzepatide	
	H3-receptor antagonist/inverse agonist	Pitolisant	
Restless leg syndrome	Dopamine agonists	Ropinirole, rotigotine	Lv et al. 2021; Winkelman et al. 2016; Silber et al. 2021
	α2δ ligands	Pregabalin, gabapentin enacarbil	
	Iron treatment	Oral ferrous sulphate	
	Low potency opioids	Tramadol, codeine	
Narcolepsy	Norepinephrine and dopamine reuptake inhibitors	Methylphenidate, solriamfetol	Thorpy and Bogan 2020; Bhattarai and Sumerall 2017; Maski et al. 2021
	Dopamine reuptake inhibitors	Modafinil, armodafinil	
	Selective serotonin and norepinephrine reuptake inhibitors	Venlafaxine	
	H3-receptor antagonist/inverse agonist	Pitolisant	
	GABA-B receptor agonist	Sodium oxybate	

## Natural remedies for sleep disorders

Using natural remedies for the treatment of sleep disorders is a common practice in modern day (Sánchez-Ortuño et al. 2009). Many of these natural remedies are food supplements consisting of different plant extracts taken for improving sleep, and are believed to be generally safe and well tolerated by the population (Guadagna et al. 2020). Many natural remedies have been studied in the management of sleep disorders such as kava (*Piper methysticum*), valerian (*Valeriana officinalis*), and passionflower (*Passiflora incarnata*) (Ekor et al. 2013). In this section, examples of natural remedies that have been reported to have potential in the management of sleep disorders are covered and discussed.

### *Artemisia* sp.

*Artemisia annua *L. (sweet wormwood or Qinghao) has an ethnomedicinal use of as sedative agent. The chloroform fraction of the methanol extract of *Artemisia annua* was found to have sedative effects in mice when injected intraperitoneally, suggested to be mediated via benzodiazepine receptors pathways (Emadi et al. 2011).

*Anthemis arvensis* (field chamomile) has been reported to be among the most cited Italian plants used for managing sleep disorders although no mechanism of action studies have been carried out yet (Motti and Falco 2021).

Interestingly, de novo formation of benzodiazepines in the plant tissue extract of *Artemisia dracunculus* (estragon) has been reported, with a binding activity to the central human benzodiazepine receptor of IC_50_ 5.7 mg/ml. The reported benzodiazepine in *Artemisia dracunculus were* delorazepam and temazepam, and their amounts ranged from 100 to 200 ng/g cell tissue (Kavvadias et al. 2000).

### *Citrus aurantium* (bitter orange)

In traditional medicine, the *Citrus genus* is used in managing the symptoms of anxiety or insomnia. The essential oil of *Citrus aurantium* L., with its main active components reported to be monoterpene limonene (98.66%), β-pinene (0.41%), and β-myrcene (0.53%), was found to exhibit anxiolytic-like activity mediated by 5-HT_1 A_-receptors in mice after acute treatment, helping in increasing sleep duration(Costa et al. 2013; de Moraes Pultrini et al. 2006; Carvalho-Freitas and Costa 2002).

In addition to *C*. *aurantium*, the sedative and anxiolytic-like effects of the essential oils of *C*. *latifolia*, and *C*. *reticulata* in mice have similarly been reported (Gargano et al. 2008).

### *Crataegus monogyna* (hawthorn)

Based on the reported traditional uses of hawthorn for its neurosedative activity, the effect of the fruit extract of hawthorn was investigated in mice and was found to have CNS depressant activities (Can et al. 2010). Hawthorn extract was also found to effectively reduce anxiety symptoms in mice (Mandanizadeh et al. 2018). In a randomized controlled trial, hawthorn fruit extract was found to significantly improve the quality of sleep in hypertensive patients when taken as a supplementary medication(Abbasi et al. 2021).

### *Eschscholzia californica* (Californian poppy)

*Eschscholzia californica* is known to have sedative and anxiolytic effects. Its main compounds are alkaloids such as protopine, californidine, allocryptopine, eschscholtzine, sanguinarine, chelerythrine, reticuline, N-methyllaurotetanine, and caryachine. Based on in vitro studies, alkaloids present in *E. californica* are suggested to act at the GABA_A_ receptors in the brain mainly at the inhibitory interneurons(Fedurco et al. 2015).

### *Humulus lupulus* (hop)

Hops oil was found to potentiate the GABA_A_ receptor response elicited by GABA (Aoshima et al. 2006). *Humulus lupulus* CO_2_ extract was found to increase the pentobarbital sleep-enhancing property (Zanoli et al. 2005), and both the ethanolic and CO_2_ extracts showed a central sedating effect (Schiller et al. 2006). Additionally, a combination of both valerian and hops extract was suggested to achieve it sleep-aiding activity possibly through interaction with melatonin and serotonin receptors (Abourashed et al. 2004). Xanthohumol, a prenylated chalcone derivative extracted from *Humulus lupulus,* was found to achieve its activity by acting at the GABA_A_ receptors (Abourashed et al. 2004).

### *Piper methysticum* (kava-kava)

Although kava-kava has been shown to have anxiolytic and hypnotic activity suggested to be through acting on GABA in animal experiments, it is not commonly prescribed or used in humans due to its hepatotoxic effects (Guadagna et al. 2020; Jussofie et al. 1994).

### *Laurus nobilis* (bay laurel)

Bay laurel was found to have sedative properties due to its content of phenylpropanoids such as eugenol and methyl eugenol and the monoterpenoid 1,8-cineole; however, mechanisms of actions or clinical data supporting their sleep-inducing activity have not been documented (Sayyah et al. 2002; Santos and Rao 2000).

### *Lavandula angustifolia* (lavender)

The main components of lavender are linalool and linalyl acetate (Guadagna et al. 2020). These molecules interact with NMDA receptors, block serotonin transporter (SERT), and lower voltage-operated calcium channels (VOOCs) (Motti and Falco 2021). Supported by preclinical studies and clinical data, lavender has shown anxiolytic and sedative properties, implying possible use in enhancing the quality of sleep (Guadagna et al. 2020; Woelk and Schläfke 2010; Kasper et al. 2010; Kasper et al. 2015; Uehleke et al. 2012).

### *Magnolia* sp.

*Magnolia* species, particularly through their bioactive compounds magnolol and honokiol, have been shown to induce REM sleep by modulating GABA_A_ receptors. These effects have been observed in in vitro studies and intraperitoneal administration in animal models, indicating their efficacy in enhancing sleep (Alexeev et al. 2012; Qu et al. 2012; Squires et al. 1999).

### *Matricaria chamomilla* (chamomile)

Chamomile has shown potential as a natural remedy for sleep disorders, supported by preclinical and clinical studies (Amsterdam et al. 2009; Mao et al. 2016). Apigenin, a flavonoid found in chamomile, was found to act as a ligand for benzodiazepine (BZD) receptors, demonstrating benzodiazepine-like activity (Medina et al. 1998), inhibiting glutamate decarboxylase (GluAD) activity, and leading to sedative and anxiolytic effects (Viola et al. 1995; Avallone et al. 2000; Awad et al. 2007; Zanoli et al. 2000).

### *Melissa officinalis* (lemon balm)

The polyphenolic content of Lemon balm was found to inhibit GABA transaminase, resulting in increased GABA levels in the brain (Awad et al. 2007; Yoo et al. 2011). This mechanism underlies its anxiolytic and sleep-enhancing properties(Kennedy et al. 2004). Both clinical and animal studies support the use of lemon balm in improving sleep quality (Kennedy et al. 2004; Cases et al. 2011; Awad et al. 2009).

### *Moringa oleifera* (drumstick tree)

The key compounds in Moringa oleifera, oleic acid, β-sitosterol, and stigmasterol have been associated with improved sleep quality via GABA_A_ receptor activity, as demonstrated in oral administration studies in animal models (Liu et al. 2020).

### *Nelumbo nucifera* (lotus)

The alkaloid fraction of the extract of lotus leaves was found to have sedative–hypnotic effects (Yan et al. 2015a). Lotus leaves alkaloids, such as nuciferine, were found to promote sleep through interactions with GABA_A_ receptors (Yan et al. 2015b).

### *Ocimum basilicum* (basil)

Basil leaves, containing monoterpenoids including linalool and phenylpropanoids such as eugenol, are traditionally used for its sedative properties (Hirai and Ito 2019). Both the volatile oil and the hydroalcoholic extract of basil leaves were found to have anxiolytic and sedative effect in mice, suggested to be due to their phenolic content (Rabbani et al. 2015).

### *Papaver rhoeas* (corn poppy)

The flavonol hyperoside is one of the main primary compounds identified in corn poppy. While it has been found to have anxiolytic and sedative effects, evidence specifically relating to sleep induction is limited (Grauso et al. 2021; Hillenbrand et al. 2004).

### *Papaver somniferum* (opium poppy)

Opium poppy, which contains alkaloids such as morphine, codeine, and noscapine, is known to modulate opioid μ-receptors (Labanca et al. 2018). These opioid compounds are associated with sedative effects, but their use is restricted due to potential for dependency and abuse (Listos et al. 2019).

### *Passiflora incarnata* (passionflower)

Passionflower has been used traditionally to treat a range of sleep disorders (Ekor et al. 2013; Bruni et al. 2021). Passionflower extract was reported to contain alkaloids and flavones that interact with GABA_A_, GABA_B_, and possibly GABA_C_ receptors, reducing sleep latency and increasing sleep duration, as supported by in vitro and animal studies (Appel et al. 2011; Elsas et al. 2010).

### *Polygala tenuifolia* (Yuan Zhi)

Tenufolin, the active compound in the well-known anti-insomnia herb *Polygala tenuifolia,* was found to enhance GABA and GABA transporter levels, resulting in prolonged sleep duration. These effects have been observed in studies using zebrafish and rats (Chen et al. 2020; Ren et al. 2020).

### *Rosmarinus officinalis* (rosemary)

Rosemary extract, rich in rosmarinic acid, caffeic acid, and flavonoids such as cirsimaritin, was found to have sleep-inducing activity via mediation of GABA_A_ receptors and inhibition of T-type calcium channels (Abdelhalim et al. 2015; Alaoui et al. 2017).

### *Schisandra chinensis* (Chinese magnolia-vine)

*Schisandra chinensis* contains the active lignan schisandrin B and schizandrin, which show notable sedative and hypnotic effects via altering the GABAergic system, raising the GABA/Glu ratio and upregulating GABA_A_ receptor subunits (Rα1 and Rγ2) in the cerebral cortex, hippocampal, and hypothalamus, yielding lower sleep latency and longer sleep duration (Li et al. 2018; Wang et al. 2020). Furthermore, schizandrin was found to reduce locomotor activity and improve pentobarbital-induced sleep patterns (Zhang et al. 2014).

### *Tilia platyphyllos* (large-leaved lime)

*Tilia platyphyllos* extract, rich in flavonoids including quercetin and rutin, was found to exhibit GABA-like and benzodiazepine-like activity. These effects were found to be mediated through modulation of GABAergic and serotonergic systems (Aguirre-Hernández et al. 2016; Allio et al. 2015; Cavadas et al. 1997).

### *Valeriana officinalis* (valerian)

Valerian is well-known for its sleep-inducing activity and is extensively used as a hypnotic and calming agent. Valerian sesquiterpenes, such as valerenic acid and valerenol, have been found to modulate GABA_A_ receptors and serotonergic systems (Benke et al. 2009; Dietz et al. 2005; Khom et al. 2007; Mineo et al. 2017). Valerian was found to significantly reduce sleep latency and improve subjective sleep quality in both clinical and preclinical studies (Mischoulon 2018; Aliakbari and Alesaeidi 2018).

### *Withania somnifera* (Ashwagandha)

Ashwagandha contains withanolide A and withaferin A, which were found to reduce sleep latency and enhance sleep quality through interactions with GABA_A_ and GABA_C_ receptors, as demonstrated both in in vitro and clinical studies (Candelario et al. 2015; Langade et al. 2019).

### *Zizyphus jujube* (jujube)

Jujube extract, containing sanjoinine A and suanzaorentang, was found to enhance GABA synthesis and act on serotonin receptors contributing to prolonged sleep time and improved sleep quality in animal studies (Yi et al. 2007; Ma et al. 2007).

To summarize the diverse natural remedies explored in this review, Table 2 provides an overview of key phytochemicals, their mechanisms of action, and their reported effects on sleep disorders.

**Table 2:** Summary of phytochemical-based natural remedies for sleep disorders, detailing their active compounds, mechanisms of action, notable effects, and supporting study types

Plant name	Active compounds	Mechanism of action	Notable effects	Study type
*Artemisia annua*	Benzodiazepines	Acts on benzodiazepine receptors	Sedative effects in mice	Animal studies
*Citrus aurantium*	Limonene, β-pinene, β-myrcene	Anxiolytic via 5-HT_1 A_ receptors	Increased sleep duration	Preclinical studies
*Crataegus monogyna*	-	CNS depressant activity	Improved sleep quality in hypertensive patients	Clinical trial
*Eschscholzia californica*	Alkaloids (e.g., protopine, sanguinarine)	Acts on GABA_A_ receptors	Sedative and anxiolytic effects	In vitro, animal studies
*Humulus lupulus*	Xanthohumol	Potentiates GABA_A_ receptor response	Enhanced pentobarbital sleep	Preclinical studies
*Laurus nobilis*	Eugenol, methyl eugenol, 1,8-cineole	-	Sedative properties	Preclinical studies
*Lavandula angustifolia*	Linalool, linalyl acetate	Interacts with NMDA receptors, blocks SERT	Anxiolytic and sedative properties	Clinical and preclinical studies
*Magnolia sp.*	Magnolol, honokiol	Modulates GABA_A_ receptors	Induces REM sleep	Animal studies
*Matricaria chamomilla*	Apigenin	Benzodiazepine receptor ligand	Sedative and anxiolytic effects	Clinical and preclinical studies
*Melissa officinalis*	Polyphenols	Inhibits GABA transaminase	Improved sleep quality	Clinical and preclinical studies
*Moringa oleifera*	Oleic acid, β-sitosterol, stigmasterol	Acts on GABA_A_ receptors	Improved sleep quality in animal models	Animal studies
*Nelumbo nucifera*	Nuciferine	Acts on GABA_A_ receptors	Sedative-hypnotic effects	Preclinical studies
*Ocimum basilicum*	Linalool, eugenol	-	Sedative and anxiolytic effects	Animal studies
*Passiflora incarnata*	Alkaloids, flavones	Acts on GABA_A_, GABA_B_, and GABA_C_ receptors	Reduced sleep latency, increased duration	In vitro, animal studies
*Polygala tenuifolia*	Tenufolin	Enhances GABA and GABA transporter levels	Prolonged sleep duration	Animal studies
*Schisandra chinensis*	Schisandrin B, schizandrin	Modulates GABAergic system, raises GABA/Glu ratio	Prolonged sleep duration, improved patterns	Animal studies
*Tilia platyphyllos*	Flavonoids (e.g., quercetin, rutin)	Modulates GABAergic and serotonergic systems	GABA-like activity	Preclinical studies
*Valeriana officinalis*	Valerenic acid, valerenol	Modulates GABA_A_ receptors and serotonergic systems	Improved sleep quality, reduced latency	Clinical and preclinical studies
*Withania somnifera*	Withanolide A, withaferin A	Acts on GABA_A_ and GABA_C_ receptors	Enhanced sleep quality	Clinical and preclinical studies
*Zizyphus jujube*	Sanjoinine A, suanzaorentang	Enhances GABA synthesis, acts on serotonin receptors	Prolonged sleep time	Animal studies

## Mechanisms of action of natural products in sleep disorders

Natural products have been traditionally used to aid in sleep disorders, and their importance is growing dramatically due to their lower side effects compared to conventional medications (Hu et al. 2018). These remedies act via various mechanisms: directly by altering neurotransmitter and hormonal pathways in sleep circuits and indirectly by improving sleep quality by relieving stress, inflammation, and oxidative stress. In this section, we aim to investigate into how nutraceuticals aid sleep.

### Modulation of neurotransmitters

Nutraceuticals can modulate neurotransmitters that regulate the sleep–wake cycle. For instance, valerian enhances sleep quality by acting on GABA_A_ receptors and improving the availability of internal GABA by inhibiting its destruction, leading to sedative effects (Murphy et al. 2010). Magnolol, nuciferine, stigmasterol, apigenin, and tenufolin display sedative effects through the GABAergic pathway (Bruni et al. 2021). Moreover, the sedative effect of chamomile extract could be justified, at least in part, by modulating serotonin and dopamine receptors (Yeom and Cho 2024). Inline, lavender interacts with serotonin transporter, exerting a sedative impact (Lopez et al. 2017). Melatonin adjustment explains also the sedative effect of several herbal products, including tart cherry (Howatson et al. 2012), St. John’s wort (Salehi et al. 2019), and kiwifruit (Doherty et al. 2023).

### Hormonal regulation and stress reduction

Indeed, stress negatively affects individuals’ cognitive and physical performance and sleep quality (Kalmbach et al. 2018). Fortunately, herbal products regulate hormones and amend stress, significant mechanisms for addressing sleep disorders. Cortisol, a stress hormone, levels are reduced by several herbal products, including ashwagandha, lavender, and rhodiola (Burns 2023). They, among others, are known for being adaptogenic, relieving stress, and enhancing mindfulness (Tóth-Mészáros et al. 2023). Noteworthy, the sympathetic system’s over-activation provokes anxiety, which passionflower could repress with minimal side effects (Janda et al. 2020). Interestingly, a double-blinded clinical study reported the anxiolytic effect of lemon balm on patients after cardiac surgery, improving their sleep quality (Soltanpour et al. 2019).

### Anti-inflammatory and antioxidant effects

Although sleep disturbance inflames systemic inflammation (Irwin et al. 2016), the reverse is also correct, and chronic inflammation indirectly lessens sleep quality (Mullington et al. 2010). Subsequently, anti-inflammatory herbal products, including turmeric, ginger, and rosemary, can enhance sleep quality (Ghasemian et al. 2016). This favorable action can be credited to reducing the sleep onset latency and wake time, improving sleep continuity (Wirth et al. 2020).

Moreover, sleep is crucial to clear reactive oxygen species that develop through the day; subsequently, inadequate sleep could provoke oxidative stress (Shah et al. 2023). Interestingly, Hill et al. pointed to a bidirectional connection between sleep and oxidative stress (Hill et al. 2018). This outcome is further supported by a recently reported randomized clinical study in around 25000 adults that concluded reducing the risk of sleep disorder upon consuming dietary antioxidants (Jiang et al. 2024). Dietary antioxidants include polyphenols, flavonoids, vitamins, and minerals (Zujko and Witkowska 2023). Noteworthy, the dietary antioxidants effect is credited to a plethora of molecular mechanisms that eventually enhance the levels of antioxidant enzymes (Lu et al. 2010).

## Summary of clinical trials and studies

Several clinical trials investigated the effect of different plant extracts on sleep related problems, which are summarized in Table 3.

**Table 3:** The summary of clinical trials of natural products in the management of sleep disorders

Sleep disorder	Study	Study design	No. of participants	Special population	Agent(s) and dose	End point/outcome(s)	Main result(s)
Impaired sleep	Taavoni et al. 2013	Randomized, triple-blind, placebo-controlled clinical trial	**100** (50 in the intervention group, 50 in the control group)	Women undergoing menopause	Valerian/Lemon Balm capsules (160 mg/80 mg)	PSQI	The valerian/lemon balm combination improved sleep quality compared to control
Impaired sleep	Adib-Hajbaghery and Mousavi 2017	Randomized controlled trial	**60** (30 in the intervention group, 30 in the control group)	Older adults	Chamomile extract capsules (200 mg, twice daily)	PSQI	8-week administration of chamomile extract can significantly improve the quality of sleep in elderly patients
Impaired sleep	Haybar et al. 2018	Randomized, Double-blind placebo-controlled clinical trial	**73** (35 in the intervention group, 38 in the control group)	Patients with chronic stable angina	Lemon balm “*Melissa officinalis*” dried aerial parts (3 g)	PSQI DASS- 21	Consumption of *Melissa officinalis* can improve depression, anxiety, stress, and insomnia in patients with chronic stable angina
Impaired sleep	Feyzabadi et al. 2018	Randomized, double-blind, placebo-controlled study	**75** (25 in the Violet oil group, 25 in the Almond oil group, 25 in the control group)	-	Violet Oil (Intranasal drops)	PSQI ISI	Significant improvement in insomnia was noticed across the 3 groups with the Violet Oil intervention being more significant
Impaired sleep	Umigai et al. 2018	Randomized, double-blind, placebo-controlled, cross-over study	**30**	-	Crocetin (7.5 mg)	OSA-MA EEG	Study participants reported improvement in sleepiness on rising and fatigue recovery (subjective sleep parameters) EEG data showed increased delta power during REM sleep latency which enhances sleep maintenance
Impaired sleep	Um et al. 2019	Randomized, double-blind, placebo-controlled, polysomnographic study	**50** (25 in the intervention group, 25 in the control group)	-	Rice Bran Extract Supplement (1000 mg)	PSQI ESS FSS SE TST WASO TWT	Rice bran extract supplement may improve sleep onset and sleep maintenance in patients with impaired sleep
Impaired sleep	Ha, et al. 2019	Randomized, double-blind, placebo-controlled trial	**80** (40 in the intervention group, 40 in the control group)	-	*Polygonatum sibiricum* (PS) rhizome extract (500 mg)	AIS (1ry) TST SE WASO	Mild insomnia might be controlled by 4-week administration of PS rhizome extract
Impaired sleep	Taherzadeh et al. 2020	Randomized, double-dummy, double-blind placebo controlled clinical trial	**50** (25 in the intervention group, 25 in the control group)	-	Dried violets (*Viola odorata* L.), saffron (*Crocus sativus* L.) and lettuce seeds (*Lactuca sativa* L.) oil preparation for intranasal administration	ISI (1ry) PSQI	The administration of the herbal intranasal formula decreased insomnia severity and improved quality of sleep
Impaired sleep	Lopresti et al. 2020	Randomized, double-blind, placebo-controlled trial	**63** (33 in the intervention group, 30 in the control group)	-	Saffron extract (14 mg, twice daily)	ISI (1ry) RSQ PSD DASS- 21	8-week supplementation of saffron extract reduced insomnia, and improved sleep quality
Impaired sleep	Elmi, et al. 2021	Randomized, triple-blind, placebo-controlled clinical trial	**76** (38 in Coronary Artery Bypass Graft (CABG) intervention group, 38 in control group)	Patients after CABG surgery	Valerian root extract powder (530 mg)	PSQI PT/PTT	Valerian root extract improved Sleep quality with no effect on coagulation profile
Impaired sleep	Shirazi, et al. 2021	Randomized, double-blind, placebo-controlled clinical trial	**60** (20 in the *Melissa officinalis L.* group, 20 in Citalopram group, 20 in the control group*)*	Postmenopausal women	lemon balm leaf and fennel fruit capsule (500 mg)	Changes in MENQOL domains	*Melissa officinalis L.* can improve the quality of life of postmenopausal women with sleep disturbances compared to other groups
Impaired sleep	Lopresti et al. 2021	Randomized double-blind placebo-controlled multi-dose study	**120** (40 in 14 mg saffron extract group, 40 in 28 mg saffron extract group, 40 in the control group)	-	Saffron extract (14 mg, 28 mg)	PSD (1ry) ISQ-W FOSQ- 10 POMS-A Salivary Cortisol Salivary Melatonin	Saffron extract can improve sleep quality and mood after awakening in addition to increasing melatonin levels
Impaired sleep	Pachikian, et al. 2021	Randomized double-blind placebo-controlled multi-dose study	**66** (32 in the intervention group, 34 in the control group)	-	Saffron extract (15.5 mg)	SOL SE TIB FRAGI TST WASO LSEQ PSQI SF- 36	Saffron extract supplementation for 6 weeks improved sleep quality-related parameters when assessed by actigraphy or questionnaires
Impaired sleep	Langade et al. 2021	Randomized, parallel, double-blind, controlled clinical trial	**80** 40 healthy subjects (20 in the intervention group, 20 in the control group) 40 patients with insomnia (20 in the intervention group, 20 in the control group)	-	Ashwagandha (*Withania somnifera* (L.) Dunal.) root extract (300 mg)	SOL TST WASO TIB SE PSQI	8-week consumption of ashwagandha root extract improved various parameters of sleep quality in healthy and insomnia patients
Impaired sleep	Karimi et al. 2023	Randomized triple-blind placebo-controlled trial	**60** (30 in the intervention group, 30 in the control group)	menopausal women	*Ocimum basilicum* leaf extract (250 mg)	PSQI III	O. basilicum leaf extract improved sleep quality and reduced the severity of insomnia in the study participants
Impaired sleep	Gutiérrez-Romero et al. 2024	Randomized, placebo-controlled trial	**64** (31 in intervention group, 27 control group)	-	Nutraceutical Formulation (green tea, lemon balm, valerian, and saffron extracts)	SE (1ry) PSQI (2ry) WASO (2ry) Salivary Cortisol (2ry) SF- 36 (2ry)	No significant effect on sleep efficiency or quality compared to placebo
Impaired sleep	Chandra Shekhar et al. 2024	Randomized, double-blind, placebo-controlled study	**80** (40 in intervention group, 40 in control group)	-	Valerian root extract (200 mg with 2% total valerenic acid)	PSQI (1ry) SL (1ry) SE (2ry) ESS (2ry) BAI (2ry) VAS (2ry)	Significant improvement in sleep quality, sleep efficiency, sleep latency and total sleep time
Impaired sleep	Pierro, et al. 2024	Randomized double-blind, placebo-controlled, and cross-over study	**30** (14 in the intervention group, 16 in the control group)	-	Lemon balm “*Melissa officinalis*” Phytosome™	Changes in ISI Sleep Quality Parameters	Melissa officinalis extract improved ISI score and extended deep sleep duration
Impaired sleep	Uchida et al. 2024	Randomized, parallel, double-blind, controlled clinical trial	**99** (49 in the intervention group, 50 in the control group)	Older Adults with cognitive decline	Match green tea capsules (2 g)	MoCA-J (1ry) ADCS-MCI-ADL (1ry) Change in PSQI (2ry)	12 months consumption of matcha green tea improved emotional perception and sleep quality
Impaired sleep	Dehghan et al. 2024	Randomized controlled clinical trial	**60** (30 in the intervention group, 30 in the control group)	Mothers of infants admitted to the neonatal intensive care unit (NICU)	Bitter orange blossom distillate syrup	STAI General Sleep Disorder Scale	The intervention had no significantly different effect on the participants’ anxiety but improved their sleep disorder state
Impaired sleep	Can et al. 2024	Randomized placebo-controlled clinical trial	**63** (21 in the Lavender group, 21 in the rosemary, 21 in the control group)	Older adults with type 2 diabetes	Lavender oil Rosemary oil (for aromatherapy)	BOMCT PSQI STAI	Aromatherapy improved quality of sleep and cognitive functions of the participants while decreasing anxiety
Impaired sleep	Kavuran and Yurttaş 2024	Randomized controlled trial	**66** (33 in the intervention group, 33 in the control group)	Patients with multiple sclerosis (MS)	Lavender oil (for aromatherapy)	FSS PSQI	Aromatherapy improved quality of sleep and reduced fatigue in patients with MS
Impaired sleep	Pérez-Piñero et al. 2024	Randomized double-blind Placebo-controlled study	**71** (33 in the intervention group, 38 in the control group)	-	extract of lemon verbena (*Aloysia citrodora*) capsule (400 mg)	VAS (1ry) SL SE PSQI PSS STAI Plasma cortisol Nocturnal melatonin	The intervention significantly improved sleep quality and elevated nocturnal melatonin levels in participating individuals
Impaired sleep	Xiong et al. 2024	Randomized triple-blind parallel-group placebo-controlled trial	**116** (96 in the intervention group, 20 in the control group)	-	Prescription of Chinese Herbal Medicine	TST (1ry) SOL WASO SE PSQI BDI SAS	Chinese medicine prescribed based on symptom differentiation can improve quality of sleep and total sleep time in patients with insomnia
Impaired sleep	Lucena et al. 2024	Randomized double-blind controlled study	**35** (17 in the intervention group, 18 in the control group)	Postmenopausal women	Lavender oil (for aromatherapy)	ESS Changes in MENQOL domains SOL TST SE	Aromatherapy improved the total sleep time, sleep efficiency and quality of life of the participants with no effect on daytime sleepiness
Impaired Sleep	Yildirim et al. 2025	Randomized, parallel, single-blind, controlled clinical trial	**100** (50 in the intervention group, 50 in the control group)	Patients with hematological malignancies	lavender oil (For aromatherapy)	Changes in RCSQ domains Changes in PFS	Aromatherapy with Lavender oil improved sleep quality and reduced fatigue levels
RLS	Cuellar and Ratcliffe 2009	Randomized, triple-blind, placebo-controlled clinical trial	**37** (17 in the intervention group, 20 in the control group)	-	Valerian capsules (800 mg)	PSQI ESS International RLS Symptom Severity Scale	Valerian capsules improved the sleep quality and RLS symptoms
RLS	Hajizadeh et al. 2023	Randomized, cross-over clinical trial	**40**	Hemodialysis patients	Valeriana officinalis L. Capsules (530 mg) compared to Gabapentin	RLS Score	Both agents were able to treat RLS with Gabapentin being more effective Both agents improved sleep quality

*SE*, sleep efficiency; *PSQI*, Pittsburgh sleep quality score; *WASO*, wake after sleep onset; *SF- 36*, 36-item short form survey score; *SL*, sleep latency; *ESS*, Epworth Sleepiness Scale; *BAI*, Beck Anxiety Inventory; *VAS*, Visual Analogue Scale; *ISI*, insomnia severity index; *DASS- 21*, Depression; Anxiety and Stress Scale – 21 items; *MENQOL*, Menopause-Specific Quality of Life Questionnaire; *RCSQ*, Richards-Campbell Sleep Questionnaire; *PFS*, Piper Fatigue Scale; *MoCA*-*J*, Montreal Cognitive Assessment-Japanese version; *ADCS-MCI-ADL*, Alzheimer’s Disease Cooperative Study Activity of Daily Living; *STAI*, Spielberger State and Trait Anxiety Inventory; *BOMCT*, Blessed Orientation Memory Concentration Test; *PSS*, Perceived Stress Scale; *FSS*, Fatigue Severity Scale; *TST*, total sleep time; *SOL*, sleep onset latency; BDI, Beck Depression Inventory; *SAS*, Self-Rating Anxiety Scale score; *SNSB*, Seoul Neuropsychological Screening Battery; *SGDS*, Short-Form Geriatric Depression Scale; *III*, insomnia intensity score; *ISQ*, Insomnia Symptom Questionnaire; *PSD*, Pittsburgh Sleep Diary; *RSQ-W*, Restorative Sleep Questionnaire- Weekly version; *FOSQ- 10*, Functional Outcomes of Sleep Questionnaire; *POMS-A*, Profile of Moods States – Abbreviated Version; *LSEQ*, The Leeds Sleep Evaluation Questionnaire; *TIB*, time in bed; FRAGI, Fragmentation Index; *HAM-A*, Hamilton Anxiety scale-A questionnaire; *RSQ*, Restorative Sleep Questionnaire; *TWT*, total wake time; *AIS*, Athens Insomnia Scale; *OSA-MA*, Oguri–Shirakawa–Azumi Sleep Inventory; Middle-age and Aged version; *EEG*, electroencephalogram

## Conclusions

Natural remedies are commonly used as sleep aids. It is frequently coupled with a general health-promoting lifestyle and may represent the widespread belief that natural goods are always excellent for sleep without risks. Kava-kava (*Piper methysticum*), Valerian (*Valerinana officinalis*), Passionflower (*Passiflora incameta*), and other herbs used to cure insomnia have gained popularity as alternative medicines. Natural products that have been demonstrated to be useful for sleeping disorders. However, preclinical and clinical investigations are required to determine the specific effects of natural compounds in the treatment of insomnia.

## Data Availability

All source data for this work (or generated in this study) are available upon reasonable request.

## References

[CR1] Abbasi A et al (2021) A comprehensive review of obstructive sleep apnea. Sleep Sci 14(2):142–154 34381578 10.5935/1984-0063.20200056 PMC8340897

[CR2] Abbasi M et al (2021) Efficacy of Hawthorn fruit extract on blood pressure and quality of sleep in patients with hypertension along with sleep disorders: a randomized double-blind controlled trial. J Contemp Med Sci 7(4):196–201

[CR3] Abdelhalim A et al (2015) Antidepressant, anxiolytic and antinociceptive activities of constituents from Rosmarinus officinalis. J Pharm Pharm Sci 18(4):448–459 26626245 10.18433/j3pw38

[CR4] Abourashed EA, Koetter U, Brattström A (2004) In vitro binding experiments with a Valerian, Hops and their fixed combination extract (Ze91019) to selected central nervous system receptors. Phytomedicine. 11(7–8):633–8 15636177 10.1016/j.phymed.2004.03.005

[CR5] Adib-Hajbaghery M, Mousavi SN (2017) The effects of chamomile extract on sleep quality among elderly people: a clinical trial. Complement Ther Med 35:109–114 29154054 10.1016/j.ctim.2017.09.010

[CR6] Aguirre-Hernández E et al (2016) Anxiolytic and sedative-like effects of flavonoids from Tilia americana var. mexicana: GABAergic and serotonergic participation (Efecto ansiolítico y sedante de flavonoides de Tilia americana var. mexicana: participación GABAérgica y serotonérgica). Salud Mental 39: 37–46

[CR7] Akkaoui MA, Palagini L, Geoffroy PA (2023) Sleep immune cross talk and insomnia. Adv Exp Med Biol 1411:263–273 36949314 10.1007/978-981-19-7376-5_12

[CR8] Alexeev M et al (2012) The natural products magnolol and honokiol are positive allosteric modulators of both synaptic and extra-synaptic GABAA receptors. Neuropharmacology 62(8):2507–2514 22445602 10.1016/j.neuropharm.2012.03.002 PMC3652012

[CR9] Aliakbari F, Alesaeidi S (2018) The effectiveness of Valeriana officinalis on sleep disturbance in patients with chronic heart failure. Int J Pharm Investig 8:145

[CR10] Alkhaldi EH et al (2023) Effect of nighttime exercise on sleep quality among the general population in Riyadh, Saudi Arabia: a cross-sectional study. Cureus 15(7):e41638. 10.7759/cureus.41638 10.7759/cureus.41638 PMC10411382 37565115

[CR11] Allen MJ, Sabir S, Sharma S (2024) GABA Receptor, in StatPearls. Treasure Island (FL) ineligible companies. Disclosure: Sarah Sabir declares no relevant financial relationships with ineligible companies. Disclosure: Sandeep Sharma declares no relevant financial relationships with ineligible companies

[CR12] Allio A et al (2015) Bud extracts from Tilia tomentosa Moench inhibit hippocampal neuronal firing through GABAA and benzodiazepine receptors activation. J Ethnopharmacol 172:288–296 26144285 10.1016/j.jep.2015.06.016

[CR13] Alnawwar MA et al (2023) The effect of physical activity on sleep quality and sleep disorder: a systematic review. Cureus 15(8):e43595. 10.7759/cureus.43595 10.7759/cureus.43595 PMC10503965 37719583

[CR14] Alruwaili NW et al (2023) The effect of nutrition and physical activity on sleep quality among adults: a scoping review. Sleep Sci Pract 7(1):8

[CR15] Alzoubaidi M, Mokhlesi B (2016) Obstructive sleep apnea during rapid eye movement sleep: clinical relevance and therapeutic implications. Curr Opin Pulm Med 22(6):545–554 27583667 10.1097/MCP.0000000000000319 PMC5084837

[CR16] Amsterdam JD et al (2009) A randomized, double-blind, placebo-controlled trial of oral matricaria recutita (chamomile) extract therapy for generalized anxiety disorder. J Clin Psychopharmacol 29(4):378–382 19593179 10.1097/JCP.0b013e3181ac935c PMC3600416

[CR17] Andrillon T, Oudiette D (2023) What is sleep exactly? Global and local modulations of sleep oscillations all around the clock. Neurosci Biobehav Rev 155:105465 37972882 10.1016/j.neubiorev.2023.105465

[CR18] Anghel L et al (2023) Sleep disorders associated with neurodegenerative diseases. Diagnostics (Basel) 13(18):2898 37761265 10.3390/diagnostics13182898 PMC10527657

[CR19] Antelmi E et al (2024) Sensory aspects of restless legs syndrome: clinical, neurophysiological and neuroimaging prospectives. Sleep Med Rev 76:101949 38749362 10.1016/j.smrv.2024.101949

[CR20] Aoshima H et al (2006) Effects of beer and hop on ionotropic γ-aminobutyric acid receptors. J Agric Food Chem 54(7):2514–2519 16569037 10.1021/jf051562a

[CR21] Appel K et al (2011) Modulation of the γ-aminobutyric acid (GABA) system by Passiflora incarnata L. Phytother Res 25(6):838–843 21089181 10.1002/ptr.3352

[CR22] Arab A et al (2023) The role of magnesium in sleep health: a systematic review of available literature. Biol Trace Elem Res 201(1):121–128 35184264 10.1007/s12011-022-03162-1

[CR23] Arendt J, Aulinas A (2000) Physiology of the pineal gland and melatonin. In: Feingold KR et al (ed) Endotext. South Dartmouth (MA) 31841296

[CR24] Arredondo E et al (2022) Overview of the role of pharmacological management of obstructive sleep apnea. Medicina (Kaunas) 58(2):225 35208549 10.3390/medicina58020225 PMC8874508

[CR25] Avallone R et al (2000) Pharmacological profile of apigenin, a flavonoid isolated from Matricaria chamomilla. Biochem Pharmacol 59(11):1387–1394 10751547 10.1016/s0006-2952(00)00264-1

[CR26] Awad R et al (2007) Effects of traditionally used anxiolytic botanicals on enzymes of the γ-aminobutyric acid (GABA) systemThis article is one of a selection of papers published in this special issue (part 1 of 2) on the Safety and Efficacy of Natural Health Products. Can J Physiol Pharmacol 85(9):933–942 18066140 10.1139/Y07-083

[CR27] Awad R et al (2009) Bioassay-guided fractionation of lemon balm (Melissa officinalis L.) using an in vitro measure of GABA transaminase activity. Phytother Res 23(8):1075–1081 19165747 10.1002/ptr.2712

[CR28] Badr MS, Dingell JD, Javaheri S (2019) Central sleep apnea: a brief review. Curr Pulmonol Rep 8(1):14–21 31788413 10.1007/s13665-019-0221-z PMC6883649

[CR29] Basit H, Damhoff TC, Huecker MR (2025) Sleeplessness and circadian disorder, in StatPearls. StatPearls Publishing Copyright © 2025, StatPearls Publishing LLC.: Treasure Island (FL) ineligible companies. Disclosure: Thomas Damhoff declares no relevant financial relationships with ineligible companies. Disclosure: Martin Huecker declares no relevant financial relationships with ineligible companies 30480971

[CR30] Basso JC, Suzuki WA (2017) The effects of acute exercise on mood, cognition, neurophysiology, and neurochemical pathways: a review. Brain Plast 2(2):127–152 29765853 10.3233/BPL-160040 PMC5928534

[CR31] Benke D et al (2009) GABAA receptors as in vivo substrate for the anxiolytic action of valerenic acid, a major constituent of valerian root extracts. Neuropharmacology 56(1):174–181 18602406 10.1016/j.neuropharm.2008.06.013

[CR32] Berridge CW, Schmeichel BE, Espana RA (2012) Noradrenergic modulation of wakefulness/arousal. Sleep Med Rev 16(2):187–197 22296742 10.1016/j.smrv.2011.12.003 PMC3278579

[CR33] Bhattarai J, Sumerall S (2017) Current and future treatment options for narcolepsy: a review. Sleep Sci 10(1):19–27 28966734 10.5935/1984-0063.20170004 PMC5611768

[CR34] Bidaki R et al (2012) A review on genetics of sleep disorders. Iran J Psychiatry Behav Sci 6(1):12–19 24644464 PMC3939950

[CR35] Bjorness TE, Greene RW (2009) Adenosine and sleep. Curr Neuropharmacol 7(3):238–245 20190965 10.2174/157015909789152182 PMC2769007

[CR36] Bonnet MH, Arand DL (1998) Heart rate variability in insomniacs and matched normal sleepers. Psychosom Med 60(5):610–615 9773766 10.1097/00006842-199809000-00017

[CR37] Bramich S et al (2023) REM sleep behaviour disorder: the importance of early identification in primary care. Br J Gen Pract 73(726):40–42 36543550 10.3399/bjgp23X731721 PMC9799336

[CR38] Briguglio M et al (2020) Healthy eating, physical activity, and sleep hygiene (HEPAS) as the winning triad for sustaining physical and mental health in patients at risk for or with neuropsychiatric disorders: considerations for clinical practice. Neuropsychiatr Dis Treat. 16:55–70 32021199 10.2147/NDT.S229206 PMC6955623

[CR39] Briskey D et al (2024) Wild Nutrition’s food-Grown® magnesium supplementation increases sleep quality and sleep duration and reduces stress in a healthy adult population: a double-blind, randomised, placebo-controlled study. Food Nutr Sci 15(7):509–523

[CR40] Bruni O et al (2021) Herbal remedies and their possible effect on the GABAergic system and sleep. Nutrients 13(2):530 33561990 10.3390/nu13020530 PMC7914492

[CR41] Bruni O et al (2021) Herbal remedies and their possible effect on the GABAergic system and sleep. Nutrients 13(2):530 33561990 10.3390/nu13020530 PMC7914492

[CR42] Brzecka A et al (2020) The association of sleep disorders, obesity and sleep-related hypoxia with cancer. Curr Genomics 21(6):444–453 33093806 10.2174/1389202921999200403151720 PMC7536792

[CR43] Burns J (2023) Common herbs for stress: the science and strategy of a botanical medicine approach to self-care. J Interprof Educ Pract 30:100592 36530213 10.1016/j.xjep.2022.100592 PMC9737923

[CR44] Buysse DJ (2014) Sleep health: can we define it? Does it matter? Sleep 37(1):9–17 24470692 10.5665/sleep.3298 PMC3902880

[CR45] Byrne R, Sinha S, Chaudhuri KR (2006) Restless legs syndrome: diagnosis and review of management options. Neuropsychiatr Dis Treat 2(2):155–164 19412460 10.2147/nedt.2006.2.2.155 PMC2671772

[CR46] Can S et al (2024) The effect of lavender and rosemary aromatherapy application on cognitive functions, anxiety, and sleep quality in the elderly with diabetes. Explore (NY) 20(6):103033 10.1016/j.explore.2024.103033 39047346

[CR47] Can ÖD et al (2010) Effects of hawthorn seed and pulp extracts on the central nervous system. Pharm Biol 48(8):924–31. 10.3109/13880200903305500 10.3109/13880200903305500 20673180

[CR48] Candelario M et al (2015) Direct evidence for GABAergic activity of Withania somnifera on mammalian ionotropic GABAA and GABAρ receptors. J Ethnopharmacol 171:264–272 26068424 10.1016/j.jep.2015.05.058

[CR49] Carrión-Pantoja S et al (2022) Insomnia symptoms, sleep hygiene, mental health, and academic performance in Spanish university students: a cross-sectional study. J Clin Med 11(7):1989 35407597 10.3390/jcm11071989 PMC8999350

[CR50] Carvalho-Freitas MIR, Costa M (2002) Anxiolytic and sedative effects of extracts and essential oil from Citrus aurantium L. Biol Pharm Bull 25(12):1629–33 12499653 10.1248/bpb.25.1629

[CR51] Cases J et al (2011) Pilot trial of Melissa officinalis L. leaf extract in the treatment of volunteers suffering from mild-to-moderate anxiety disorders and sleep disturbances. Med J Nutr Metab 4:211–218 10.1007/s12349-010-0045-4 PMC3230760 22207903

[CR52] Castano MY et al (2019) Melatonin improves mood status and quality of life and decreases cortisol levels in fibromyalgia. Biol Res Nurs 21(1):22–29 30415563 10.1177/1099800418811634

[CR53] Castelnovo A et al (2018) NREM sleep parasomnias as disorders of sleep-state dissociation. Nat Rev Neurol 14(8):470–481 29959394 10.1038/s41582-018-0030-y

[CR54] Cavadas C et al (1997) In vitro study of the interaction of Tilia europeae L. aqueous extract with GABAA receptors in rat brain. Phytother Res 11(1):17–21

[CR55] Caylak E (2009) The genetics of sleep disorders in humans: narcolepsy, restless legs syndrome, and obstructive sleep apnea syndrome. Am J Med Genet A 149A(11):2612–2626 19876894 10.1002/ajmg.a.33087

[CR56] Chandra Shekhar H, Joshua L, Thomas JV (2024) Standardized extract of valeriana officinalis improves overall sleep quality in human subjects with sleep complaints: a randomized, double-blind, placebo-controlled, clinical study. Adv Ther. 41(1):246–261 37899385 10.1007/s12325-023-02708-6 PMC10796483

[CR57] Chang J (2000) Medicinal herbs: drugs or dietary supplements? Biochem Pharmacol 59(3):211–219 10609549 10.1016/s0006-2952(99)00243-9

[CR58] Chavda V et al (2022) Narcolepsy-a neuropathological obscure sleep disorder: a narrative review of current literature. Brain Sci 12(11):1473 36358399 10.3390/brainsci12111473 PMC9688775

[CR59] Chen ZW et al (2020) Effects of tenuifolin on rest/wake behaviour in zebrafish. Exp Ther Med 19(3):2326–2334 32104301 10.3892/etm.2020.8476 PMC7027208

[CR60] Climent-Sanz C et al (2022) Cognitive behavioral therapy for insomnia (CBT-i) in patients with fibromyalgia: a systematic review and meta-analysis. Disabil Rehabil. 44(20):5770–5783 34297651 10.1080/09638288.2021.1954706

[CR61] Corrêa CC et al (2024) Sleep hygiene intervention improves sleep time and duration in high school students. Sleep Sci 17(3):e297–e303 39268346 10.1055/s-0044-1782169 PMC11390171

[CR62] Costa CARA et al (2013) Citrus aurantium L. essential oil exhibits anxiolytic-like activity mediated by 5-HT1A-receptors and reduces cholesterol after repeated oral treatment. BMC Complement Altern Med 13:42. 10.1186/1472-6882-13-42 10.1186/1472-6882-13-42 PMC3598547 23432968

[CR63] Costello RB et al (2014) The effectiveness of melatonin for promoting healthy sleep: a rapid evidence assessment of the literature. Nutr J 13:106 25380732 10.1186/1475-2891-13-106 PMC4273450

[CR64] Cuellar NG, Ratcliffe SJ (2009) Does valerian improve sleepiness and symptom severity in people with restless legs syndrome? Altern Ther Health Med 15(2):22–28 19284179

[CR65] de Moraes Pultrini A, Almeida Galindo L, Costa M (2006) Effects of the essential oil from Citrus aurantium L in experimental anxiety models in mice - PubMed. Life Sci 78(15):1720–5 16253279 10.1016/j.lfs.2005.08.004

[CR66] Dehghan Z et al (2024) Effect of bitter orange blossom distillate on anxiety and sleep disorder in mothers with infants admitted to neonatal intensive care unit: a randomized controlled clinical trial. PLoS ONE 19(8):e0306887 39133687 10.1371/journal.pone.0306887 PMC11318878

[CR67] Di Pierro F et al (2024) Effects of Melissa officinalis phytosome on sleep quality: results of a prospective, double-blind, placebo-controlled, and cross-over study. Nutrients 16(23):4199 39683592 10.3390/nu16234199 PMC11644815

[CR68] Dietz BM et al (2005) Valerian extract and valerenic acid are partial agonists of the 5-HT5a receptor in vitro. Mol Brain Res 138(2):191–197 15921820 10.1016/j.molbrainres.2005.04.009 PMC5805132

[CR69] Doghramji K (2007) Melatonin and its receptors: a new class of sleep-promoting agents. J Clin Sleep Med 3(5 Suppl):S17-23 17824497 PMC1978320

[CR70] Doherty R et al (2023) The impact of kiwifruit consumption on the sleep and recovery of elite athletes. Nutrients 15(10):2274 37242157 10.3390/nu15102274 PMC10220871

[CR71] Dubocovich ML (2007) Melatonin receptors: role on sleep and circadian rhythm regulation. Sleep Med 8(Suppl 3):34–42 18032103 10.1016/j.sleep.2007.10.007

[CR72] Duraccio KM et al (2022) Relationship of overweight and obesity to insomnia severity, sleep quality, and insomnia improvement in a clinically referred pediatric sample. J Clin Sleep Med 18(4):1083–1091 34879901 10.5664/jcsm.9806 PMC8974392

[CR73] Eban-Rothschild A, Appelbaum L, de Lecea L (2018) Neuronal mechanisms for sleep/wake regulation and modulatory drive. Neuropsychopharmacology 43(5):937–952 29206811 10.1038/npp.2017.294 PMC5854814

[CR74] Ekor M et al (2013) Management of anxiety and sleep disorders: role of complementary and alternative medicine and challenges of integration with conventional orthodox care. Chin J Integr Med 19(1):5–14. 10.1007/s11655-013-1197-5 10.1007/s11655-013-1197-5 23275011

[CR75] El Alaoui C et al (2017) Modulation of T-type Ca2+ channels by lavender and rosemary extracts. PLoS ONE 12(10):e0186864 29073181 10.1371/journal.pone.0186864 PMC5658086

[CR76] Elsas SM et al (2010) Passiflora incarnata L. (Passionflower) extracts elicit GABA currents in hippocampal neurons in vitro, and show anxiogenic and anticonvulsant effects in vivo, varying with extraction method. Phytomedicine 17(12):940–949 20382514 10.1016/j.phymed.2010.03.002 PMC2941540

[CR77] Emadi F et al (2011) Sedative effects of Iranian Artemisia annua in mice: possible benzodiazepine receptors involvement. Pharm Biol 49(8):784–8 21554148 10.3109/13880209.2010.548389

[CR78] Ernst E (2006) When natural is not harmless. International journal of clinical practice,Wiley Online Library, pp 380–380. 10.1111/j.1368-5031.2006.00924b.x 10.1111/j.1368-5031.2006.00924b.x 16620347

[CR79] Eugene AR, Masiak J (2015) The neuroprotective aspects of sleep. Medtube Sci 3(1):35–40 26594659 PMC4651462

[CR80] Fedurco M et al (2015) Modulatory effects of Eschscholzia californica alkaloids on recombinant GABAA receptors. Biochem Res Int. 1:617620 10.1155/2015/617620 PMC4609799 26509084

[CR81] Feige B et al (2018) Insomnia-perchance a dream? Results from a NREM/REM sleep awakening study in good sleepers and patients with insomnia. Sleep 41(5):zsy032 10.1093/sleep/zsy032 29432570

[CR82] Feyzabadi Z et al (2018) Efficacy of Violet oil, a traditional Iranian formula, in patients with chronic insomnia: A randomized, double-blind, placebo-controlled study. J Ethnopharmacol 214:22–28 29217495 10.1016/j.jep.2017.11.036

[CR83] Franceschini C et al (2021) A practical guide to the pharmacological and behavioral therapy of Narcolepsy. Neurotherapeutics 18(1):6–19 33886090 10.1007/s13311-021-01051-4 PMC8061157

[CR84] Gangwisch JE et al (2020) High glycemic index and glycemic load diets as risk factors for insomnia: analyses from the Women’s Health Initiative. Am J Clin Nutr. 111(2):429–439 31828298 10.1093/ajcn/nqz275 PMC6997082

[CR85] Gargano AC, Costa AML, Costa M (2008) Essential oils from Citrus latifolia and Citrus reticulata reduce anxiety and prolong ether sleeping time in mice. Tree and Forestry Science and Biotechnology 2 (Special Issue 1):121–124

[CR86] Gaskin CJ et al (2024) Sleep behavioral outcomes of school-based interventions for promoting sleep health in children and adolescents aged 5 to 18 years: a systematic review. Sleep 5(1):zpae019 10.1093/sleepadvances/zpae019 PMC10996385 38584765

[CR87] Ghasemian M, Owlia S, Owlia MB (2016) Review of anti-inflammatory herbal medicines. Adv Pharmacol Sci 2016:9130979 27247570 10.1155/2016/9130979 PMC4877453

[CR88] Gobbi G, Comai S (2019) Differential function of melatonin MT(1) and MT(2) receptors in REM and NREM sleep. Front Endocrinol (Lausanne) 10:87 30881340 10.3389/fendo.2019.00087 PMC6407453

[CR89] Gottesman RF et al (2024) Impact of sleep disorders and disturbed sleep on brain health: a scientific statement from the American Heart Association. Stroke 55(3):e61–e76 38235581 10.1161/STR.0000000000000453

[CR90] Gottesmann C (2002) GABA mechanisms and sleep. Neuroscience 111(2):231–239 11983310 10.1016/s0306-4522(02)00034-9

[CR91] Grauso L et al (2021) Corn poppy, Papaver rhoeas L.: a critical review of its botany, phytochemistry and pharmacology. Phytochem Rev 20(1):227–248

[CR92] Guadagna S et al (2020) Plant extracts for sleep disturbances: a systematic review. Evid Based Complement Alternat Med 2020(1):3792390 32382286 10.1155/2020/3792390 PMC7191368

[CR93] Gupta R et al (2016) Restless legs syndrome and pregnancy: prevalence, possible pathophysiological mechanisms and treatment. Acta Neurol Scand 133(5):320–329 26482928 10.1111/ane.12520 PMC5562408

[CR94] Gutiérrez-Romero SA et al (2024) Effect of a nutraceutical combination on sleep quality among people with impaired sleep: a randomised, placebo-controlled trial. Sci Rep 14(1):8062 38580720 10.1038/s41598-024-58661-z PMC10997602

[CR95] Ha E et al (2019) Efficacy of Polygonatum sibiricum on mild insomnia: a randomized placebo-controlled trial. Nutrients 11(8):1719 31349690 10.3390/nu11081719 PMC6723095

[CR96] Hajizadeh I et al (2023) Comparison the effect of valerian and gabapentin on RLS and sleep quality in hemodialysis patients: a randomized clinical trial. Ther Apher Dial 27(4):621–628 37039703 10.1111/1744-9987.13987

[CR97] Harvey AG (2002) A cognitive model of insomnia. Behav Res Ther 40(8):869–893 12186352 10.1016/s0005-7967(01)00061-4

[CR98] Haybar H et al (2018) The effects of Melissa officinalis supplementation on depression, anxiety, stress, and sleep disorder in patients with chronic stable angina. Clin Nutr ESPEN 26:47–52 29908682 10.1016/j.clnesp.2018.04.015

[CR99] Hensley JG (2009) Leg cramps and restless legs syndrome during pregnancy. J Midwifery Womens Health 54(3):211–218 19410213 10.1016/j.jmwh.2009.01.003

[CR100] Hepsomali P, Groeger JA (2021) Diet, sleep, and mental health: insights from the UK biobank study. Nutrients 13(8):2573 34444731 10.3390/nu13082573 PMC8398967

[CR101] Hernandez AB, Patil SP (2016) Pathophysiology of central sleep apneas. Sleep Breath 20(2):467–482 26782104 10.1007/s11325-015-1290-z

[CR102] Hill VM et al (2018) A bidirectional relationship between sleep and oxidative stress in Drosophila. PLoS Biol 16(7):e2005206 30001323 10.1371/journal.pbio.2005206 PMC6042693

[CR103] Hillenbrand M, Zapp J, Becker H (2004) Depsides from the petals of Papaver rhoeas. Planta Med 70(04):380–382 15095160 10.1055/s-2004-818956

[CR104] Hirai M, Ito M (2019) Sedative effects of the essential oil and headspace air of Ocimum basilicum by inhalation in mice. J Nat Med 73(1):283–288 30343352 10.1007/s11418-018-1253-3

[CR105] Hirotsu C, Tufik S, Andersen ML (2015) Interactions between sleep, stress, and metabolism: from physiological to pathological conditions. Sleep Sci 8(3):143–152 26779321 10.1016/j.slsci.2015.09.002 PMC4688585

[CR106] Howatson G et al (2012) Effect of tart cherry juice (Prunus cerasus) on melatonin levels and enhanced sleep quality. Eur J Nutr 51(8):909–916 22038497 10.1007/s00394-011-0263-7

[CR107] Hu Z et al (2018) Sleep-aids derived from natural products. Biomol Ther (Seoul) 26(4):343–349 29929351 10.4062/biomolther.2018.099 PMC6029681

[CR108] Hwang H et al (2022) Sleep state of the elderly population in Korea: nationwide cross-sectional population-based study. Front Neurol 13:1095404 36698878 10.3389/fneur.2022.1095404 PMC9868806

[CR109] Iao SI et al (2022) Associations between bedtime eating or drinking, sleep duration and wake after sleep onset: findings from the American time use survey. Br J Nutr 127(12):1888–1897 10.1017/S0007114521003597 PMC9092657 34511160

[CR110] Irwin MR, Olmstead R, Carroll JE (2016) Sleep disturbance, sleep duration, and inflammation: a systematic review and meta-analysis of cohort studies and experimental sleep deprivation. Biol Psychiatry 80(1):40–52 26140821 10.1016/j.biopsych.2015.05.014 PMC4666828

[CR111] Ishikawa O, Oks M (2021) Central sleep apnea. Clin Geriatr Med 37(3):469–481 34210451 10.1016/j.cger.2021.04.009

[CR112] Janda K et al (2020) Passiflora incarnata in neuropsychiatric disorders-a systematic review. Nutrients 12(12):3894 33352740 10.3390/nu12123894 PMC7766837

[CR113] Jansson-Fröjmark M et al (2024) Stimulus control for insomnia: a systematic review and meta-analysis. J Sleep Res 33(1):e14002 37496454 10.1111/jsr.14002

[CR114] Javaheri S et al (2017) Sleep apnea. J Am Coll Cardiol 69(7):841–858 28209226 10.1016/j.jacc.2016.11.069 PMC5393905

[CR115] Jerath R, Beveridge C, Barnes VA (2019) Self-regulation of breathing as an adjunctive treatment of insomnia. Front Psychiatry. 9:423812 10.3389/fpsyt.2018.00780 PMC6361823 30761030

[CR116] Jiang J et al (2024) Antioxidants and the risk of sleep disorders: results from NHANES and two-sample Mendelian randomization study. Front Nutr 11:1453064 39416650 10.3389/fnut.2024.1453064 PMC11480095

[CR117] Joiner WJ (2018) The neurobiological basis of sleep and sleep disorders. Physiology (Bethesda) 33(5):317–327 30109824 10.1152/physiol.00013.2018 PMC6230548

[CR118] Jordan AS, McSharry DG, Malhotra A (2014) Adult obstructive sleep apnoea. Lancet 383(9918):736–747 23910433 10.1016/S0140-6736(13)60734-5 PMC3909558

[CR119] Jussofie A, Schmiz A, Hiemke C (1994) Kavapyrone enriched extract from Piper methysticum as modulator of the GABA binding site in different regions of rat brain. Psychopharmacology 116(4):469–474 7701051 10.1007/BF02247480

[CR120] Kaczmarski P et al (2023) Influence of glutamatergic and GABAergic neurotransmission on obstructive sleep apnea. Front Neurosci 17:1213971 37521710 10.3389/fnins.2023.1213971 PMC10372424

[CR121] Kalmbach DA, Anderson JR, Drake CL (2018) The impact of stress on sleep: pathogenic sleep reactivity as a vulnerability to insomnia and circadian disorders. J Sleep Res 27(6):e12710 29797753 10.1111/jsr.12710 PMC7045300

[CR122] Kang Y, Kang X, Cai Y (2022) The gut microbiome as a target for adjuvant therapy in insomnia disorder. Clin Res Hepatol Gastroenterol 46(1):101834 34800683 10.1016/j.clinre.2021.101834

[CR123] Kansagra S (2020) Sleep disorders in adolescents. Pediatrics 145(Suppl 2):S204–S209 32358212 10.1542/peds.2019-2056I

[CR124] Karimi FZ et al (2023) The effect of oral capsule of Ocimum basilicum leaf extract on sleep quality and insomnia severity in menopausal women: a randomized clinical trial. Phytother Res 37(6):2344–2352 36750371 10.1002/ptr.7753

[CR125] Kasper S et al (2010) Silexan, an orally administered Lavandula oil preparation, is effective in the treatment of ‘subsyndromal’ anxiety disorder: a randomized, double-blind, placebo controlled trial. Int Clin Psychopharmacol 25(5):277–287 20512042 10.1097/YIC.0b013e32833b3242

[CR126] Kasper S, Anghelescu I, Dienel A (2015) Efficacy of orally administered Silexan in patients with anxiety-related restlessness and disturbed sleep–a randomized, placebo-controlled trial. Eur Neuropsychopharmacol 25(11):1960–1967 26293583 10.1016/j.euroneuro.2015.07.024

[CR127] Kavuran E, Yurttaş A (2024) The effect of aromatherapy with lavender essential oil on the sleep and fatigue level of patients with multiple sclerosis in Turkey: a randomized controlled trial. Niger J Clin Pract 27(5):635–642 38842713 10.4103/njcp.njcp_811_23

[CR128] Kavvadias D, Abou-Mandour AA, Czygan FC, Beckmann H, Sand P, Riederer P, Schreie P (2000) Identification of benzodiazepines in Artemisia dracunculus and Solanum tuberosum rationalizing Their endogenous formation in plant tissue. Biochem Biophys Res Commun. 269(1):290–5 10694515 10.1006/bbrc.2000.2283

[CR129] Kawai N et al (2015) The sleep-promoting and hypothermic effects of glycine are mediated by NMDA receptors in the suprachiasmatic nucleus. Neuropsychopharmacology 40(6):1405–1416 25533534 10.1038/npp.2014.326 PMC4397399

[CR130] Kember AJ et al (2023) Common sleep disorders in pregnancy: a review. Front Med (Lausanne) 10:1235252 37671402 10.3389/fmed.2023.1235252 PMC10475609

[CR131] Kennedy DO, Little W, Scholey AB (2004) Attenuation of laboratory-induced stress in humans after acute administration of Melissa officinalis (Lemon Balm). Psychosom Med 66(4):607–613 15272110 10.1097/01.psy.0000132877.72833.71

[CR132] Kesztyüs D et al (2021) Applicability of time-restricted eating for the prevention of lifestyle-dependent diseases in a working population: results of a pilot study in a pre-post design. Ger Med Sci 19:Doc04. 10.3205/000291 10.3205/000291 PMC8051591 33911996

[CR133] Khachatryan SG et al (2020) Sleep-related movement disorders in a population of patients with epilepsy: prevalence and impact of restless legs syndrome and sleep bruxism. J Clin Sleep Med 16(3):409–414 31992428 10.5664/jcsm.8218 PMC7075103

[CR134] Khom S et al (2007) Valerenic acid potentiates and inhibits GABAA receptors: molecular mechanism and subunit specificity. Neuropharmacology 53(1):178–187 17585957 10.1016/j.neuropharm.2007.04.018

[CR135] Kim B et al (2024) Restless legs syndrome in patients with obstructive sleep apnea: association between apnea severity and symptoms of depression, insomnia, and daytime sleepiness. Sleep Med. 117:40–45 38507975 10.1016/j.sleep.2024.03.009

[CR136] Kornum BR (2020) Narcolepsy type 1: what have we learned from immunology? Sleep 43(10):zsaa055 32227223 10.1093/sleep/zsaa055

[CR137] Kornum BR et al (2017) Narcolepsy. Nat Rev Dis Prim 3(1):16100 28179647 10.1038/nrdp.2016.100

[CR138] Kripke DF (2018) Hypnotic drug risks of mortality, infection, depression, and cancer: but lack of benefit. F1000Research 5:918 10.12688/f1000research.8729.1 PMC4890308 27303633

[CR139] Laaboub N et al (2022) Insomnia disorders are associated with increased cardiometabolic disturbances and death risks from cardiovascular diseases in psychiatric patients treated with weight-gain-inducing psychotropic drugs: results from a Swiss cohort. BMC Psychiatry 22(1):342 35581641 10.1186/s12888-022-03983-3 PMC9116036

[CR140] Labanca F, Ovesnà J, Milella L (2018) Papaver somniferum L. taxonomy, uses and new insight in poppy alkaloid pathways. Phytochem Rev 17(4):853–871

[CR141] Laborde S et al (2019) Influence of a 30-day slow-paced breathing intervention compared to social media use on subjective sleep quality and cardiac vagal activity. J Clin Med 8(2):193 30736268 10.3390/jcm8020193 PMC6406675

[CR142] Lader M (2011) Benzodiazepines revisited—will we ever learn? Addiction 106(12):2086–2109 21714826 10.1111/j.1360-0443.2011.03563.x

[CR143] Langade D et al (2019) Efficacy and safety of Ashwagandha (Withania somnifera) root extract in insomnia and anxiety: a double-blind, randomized, placebo-controlled study. Cureus 11(9):e5797 31728244 10.7759/cureus.5797 PMC6827862

[CR144] Langade D et al (2021) Clinical evaluation of the pharmacological impact of ashwagandha root extract on sleep in healthy volunteers and insomnia patients: a double-blind, randomized, parallel-group, placebo-controlled study. J Ethnopharmacol 264:113276 32818573 10.1016/j.jep.2020.113276

[CR145] Lee J et al (2019) Sleep disorders and menopause. J Menopausal Med 25(2):83–87 31497577 10.6118/jmm.19192 PMC6718648

[CR146] Li N et al (2018) Sedative and hypnotic effects of Schisandrin B through increasing GABA/Glu ratio and upregulating the expression of GABAA in mice and rats. Biomed Pharmacother 103:509–516 29677536 10.1016/j.biopha.2018.04.017

[CR147] Liblau RS et al (2024) The immunopathogenesis of narcolepsy type 1. Nat Rev Immunol 24(1):33–48 37400646 10.1038/s41577-023-00902-9

[CR148] Listos J et al (2019) The mechanisms involved in morphine addiction: an overview. Int J Mol Sci 20(17):4302 31484312 10.3390/ijms20174302 PMC6747116

[CR149] Liu JH, Park KH (1988) Gonadotropin and prolactin secretion increases during sleep during the puerperium in nonlactating women. J Clin Endocrinol Metab 66(4):839–845 3126216 10.1210/jcem-66-4-839

[CR150] Liu W-L et al (2020) Moringa oleifera lam seed oil augments pentobarbital-induced sleeping behaviors in mice via GABAergic systems. J Agric Food Chem 68(10):3149–3162 32062961 10.1021/acs.jafc.0c00037

[CR151] Liu K et al (2020) Effects of progressive muscle relaxation on anxiety and sleep quality in patients with COVID-19. Complement Ther Clin Pract 39:101132 32379667 10.1016/j.ctcp.2020.101132 PMC7102525

[CR152] Liu J et al (2024) Pharmacological interventions for the treatment of obstructive sleep apnea syndrome. Front Med (Lausanne) 11:1359461 38495117 10.3389/fmed.2024.1359461 PMC10943699

[CR153] Lopez V et al (2017) Exploring pharmacological mechanisms of lavender (Lavandula angustifolia) essential oil on central nervous system targets. Front Pharmacol 8:280 28579958 10.3389/fphar.2017.00280 PMC5437114

[CR154] Lopresti AL et al (2020) Effects of saffron on sleep quality in healthy adults with self-reported poor sleep: a randomized, double-blind, placebo-controlled trial. J Clin Sleep Med 16(6):937–947 32056539 10.5664/jcsm.8376 PMC7849671

[CR155] Lopresti AL, Smith SJ, Drummond PD (2021) An investigation into an evening intake of a saffron extract (affron®) on sleep quality, cortisol, and melatonin concentrations in adults with poor sleep: a randomised, double-blind, placebo-controlled, multi-dose study. Sleep Med 86:7–18 34438361 10.1016/j.sleep.2021.08.001

[CR156] Lu JM et al (2010) Chemical and molecular mechanisms of antioxidants: experimental approaches and model systems. J Cell Mol Med 14(4):840–860 19754673 10.1111/j.1582-4934.2009.00897.x PMC2927345

[CR157] Lu M et al (2023) Comparative effectiveness of digital cognitive behavioral therapy vs medication therapy among patients with insomnia. JAMA Netw Open 6(4):e237597–e237597 37040111 10.1001/jamanetworkopen.2023.7597 PMC10091171

[CR158] Lucena L et al (2024) Effect of a lavender essential oil and sleep hygiene protocol on insomnia in postmenopausal women: a pilot randomized clinical trial. Explore (NY) 20(1):116–125 37495431 10.1016/j.explore.2023.07.004

[CR159] Lv Q et al (2021) Pharmacologic treatment of restless legs syndrome. Curr Neuropharmacol 19(3):372–382 33380302 10.2174/1570159X19666201230150127 PMC8033969

[CR160] Lv R et al (2023) Pathophysiological mechanisms and therapeutic approaches in obstructive sleep apnea syndrome. Signal Transduct Target Ther 8(1):218 37230968 10.1038/s41392-023-01496-3 PMC10211313

[CR161] Ma Y et al (2007) Sanjoinine A isolated from Zizyphi Spinosi Semen augments pentobarbital-induced sleeping behaviors through the modification of GABA-ergic systems. Biol Pharm Bull 30(9):1748–1753 17827733 10.1248/bpb.30.1748

[CR162] Madari S et al (2021) Pharmacological management of insomnia. Neurotherapeutics 18(1):44–52 33527255 10.1007/s13311-021-01010-z PMC8116439

[CR163] Mai E, Buysse DJ (2008) Insomnia: prevalence, impact, pathogenesis, differential diagnosis, and evaluation. Sleep Med Clin 3(2):167–174 19122760 10.1016/j.jsmc.2008.02.001 PMC2504337

[CR164] Manconi M et al (2012) When gender matters: restless legs syndrome. Report of the “RLS and woman” workshop endorsed by the European RLS Study Group. Sleep Med Rev 16(4):297–307 22075215 10.1016/j.smrv.2011.08.006

[CR165] Mandanizadeh K, Modaresi M, Sajjadian I (2018) Comparing the effect of Hawthorn’s extract and chlordiazepoxide on reducing anxiety in laboratory mice. iNDO Am J Pharm Sci 5(5):4435–4440

[CR166] Manfredi RL, Brennan RW, Cadieux RJ (1987) Disorders of excessive sleepiness: narcolepsy and hypersomnia. Semin Neurol 7(3):250–258 3332460 10.1055/s-2008-1041425

[CR167] Manfridi A, Brambilla D, Mancia M (1999) Stimulation of NMDA and AMPA receptors in the rat nucleus basalis of Meynert affects sleep. Am J Physiol 277(5):R1488–R1492 10564223 10.1152/ajpregu.1999.277.5.R1488

[CR168] Mao JJ et al (2016) Long-term chamomile (Matricaria chamomilla L.) treatment for generalized anxiety disorder: a randomized clinical trial. Phytomedicine 23(14):1735–1742 27912875 10.1016/j.phymed.2016.10.012 PMC5646235

[CR169] Maski K et al (2021) Treatment of central disorders of hypersomnolence: an American Academy of Sleep Medicine clinical practice guideline. J Clin Sleep Med 17(9):1881–1893 34743789 10.5664/jcsm.9328 PMC8636351

[CR170] Maurer LF et al (2020) Isolating the role of time in bed restriction in the treatment of insomnia: a randomized, controlled, dismantling trial comparing sleep restriction therapy with time in bed regularization. Sleep 43(11):zsaa096 32421814 10.1093/sleep/zsaa096

[CR171] Maurer LF et al (2021) The clinical effects of sleep restriction therapy for insomnia: a meta-analysis of randomised controlled trials. Sleep Med Rev 58:101493 33984745 10.1016/j.smrv.2021.101493

[CR172] McArdle N et al (2020) The prevalence of common sleep disorders in young adults: a descriptive population-based study. Sleep 43(10):zsaa072 32280974 10.1093/sleep/zsaa072

[CR173] Medina JH et al (1998) Neuroactive flavonoids: new ligands for the Benzodiazepine receptors. Phytomedicine 5(3):235–243 23195847 10.1016/S0944-7113(98)80034-2

[CR174] Mineo L et al (2017) Valeriana officinalis root extract modulates cortical excitatory circuits in humans. Neuropsychobiology 75(1):46–51 29035887 10.1159/000480053

[CR175] Mischoulon D (2018) Popular herbal and natural remedies used in psychiatry. Focus 16(1):2–11 31975894 10.1176/appi.focus.20170041 PMC6519573

[CR176] Miyagawa T, Tokunaga K (2019) Genetics of Narcolepsy. Hum Genome Var 6:4 30652006 10.1038/s41439-018-0033-7 PMC6325123

[CR177] Monti JM, Jantos H (2008) The roles of dopamine and serotonin, and of their receptors, in regulating sleep and waking. Prog Brain Res 172:625–646 18772053 10.1016/S0079-6123(08)00929-1

[CR178] Morgan D, Tsai SC (2015) Sleep and the endocrine system. Crit Care Clin 31(3):403–418 26118912 10.1016/j.ccc.2015.03.004

[CR179] Morin CM et al (2015) Insomnia disorder. Nat Rev Dis Primers 1(1):1–18 10.1038/nrdp.2015.26 27189779

[CR180] Morin CM et al (2020) Incidence, persistence, and remission rates of insomnia over 5 years. JAMA Netw Open 3(11):e2018782 33156345 10.1001/jamanetworkopen.2020.18782 PMC7648256

[CR181] Motti R, de Falco B (2021) Traditional herbal remedies used for managing anxiety and insomnia in Italy: an ethnopharmacological overview. Horticulturae 7(12):523

[CR182] Mullington JM et al (2010) Sleep loss and inflammation. Best Pract Res Clin Endocrinol Metab 24(5):775–784 21112025 10.1016/j.beem.2010.08.014 PMC3548567

[CR183] Murphy K et al (2010) Valeriana officinalis root extracts have potent anxiolytic effects in laboratory rats. Phytomedicine 17(8–9):674–678 20042323 10.1016/j.phymed.2009.10.020

[CR184] Mussi N et al (2023) The first-line approach in children with obstructive sleep apnea syndrome (OSA). J Clin Med 12(22):7092 38002704 10.3390/jcm12227092 PMC10672526

[CR185] Nanayakkara B, Di Michiel J, Yee BJ (2023) Restless legs syndrome. Aust J Gen Pract 52(9):615–621 37666782 10.31128/AJGP-02-23-6722

[CR186] Ni H, Simile C, Hardy AM (2002) Utilization of complementary and alternative medicine by United States adults: results from the 1999 national health interview survey. Med Care 40(4):353–358 12021691 10.1097/00005650-200204000-00011

[CR187] Nobre ML et al (2024) Pharmacological treatment for obstructive sleep apnea: a systematic review and meta-analysis. Clinics 79:100330 38341903 10.1016/j.clinsp.2024.100330 PMC10869242

[CR188] Norouzi E et al (2024) Combined mindfulness-based stress reduction and physical activity improved psychological factors and sleep quality in patients with MDD: a randomized controlled trial study. Arch Psychiatr Nurs 53:215–223 39615937 10.1016/j.apnu.2024.10.020

[CR189] Ntikoudi A et al (2024) The effectiveness of cognitive behavioral therapy on insomnia severity among menopausal women: a scoping review. Life (Basel) 14(11):1405 39598203 10.3390/life14111405 PMC11595697

[CR190] Ohayon MM et al (2023) Prevalence and incidence of narcolepsy symptoms in the US general population. Sleep Med X 6:100095 38149177 10.1016/j.sleepx.2023.100095 PMC10749896

[CR191] Ollila HM et al (2023) Narcolepsy risk loci outline role of T cell autoimmunity and infectious triggers in narcolepsy. Nat Commun 14(1):2709 37188663 10.1038/s41467-023-36120-z PMC10185546

[CR192] Pachikian BD et al (2021) Effects of saffron extract on sleep quality: a randomized double-blind controlled clinical trial. Nutrients 13(5):1473 33925432 10.3390/nu13051473 PMC8145009

[CR193] Padgett CL, Slesinger PA (2010) GABAB receptor coupling to G-proteins and ion channels. Adv Pharmacol 58:123–147 20655481 10.1016/S1054-3589(10)58006-2

[CR194] Palagini L et al (2022) Sleep, insomnia and mental health. J Sleep Res 31(4):e13628 35506356 10.1111/jsr.13628

[CR195] Palagini L et al (2023) Potential genetic and epigenetic mechanisms in insomnia: a systematic review. J Sleep Res 32(6):e13868 36918298 10.1111/jsr.13868

[CR196] Para KS et al (2019) Suicidal thought and behavior in individuals with restless legs syndrome. Sleep Med 54:1–7 30529070 10.1016/j.sleep.2018.09.019

[CR197] Parmentier R et al (2007) The brain H3-receptor as a novel therapeutic target for vigilance and sleep-wake disorders. Biochem Pharmacol 73(8):1157–1171 17288995 10.1016/j.bcp.2007.01.002

[CR198] Patel AK et al (2024) Physiology, sleep stages, in StatPearls. Treasure Island (FL) ineligible companies. Disclosure: Vamsi Reddy declares no relevant financial relationships with ineligible companies. Disclosure: Karlie Shumway declares no relevant financial relationships with ineligible companies. Disclosure: John Araujo declares no relevant financial relationships with ineligible companies

[CR199] Pereira N et al (2020) Influence of dietary sources of melatonin on sleep quality: a review. J Food Sci 85(1):5–13 31856339 10.1111/1750-3841.14952

[CR200] Pérez-Piñero S et al (2024) Dietary supplementation with an extract of Aloysia citrodora (lemon verbena) improves sleep quality in healthy subjects: a randomized double-blind controlled study. Nutrients 16(10):1523 38794761 10.3390/nu16101523 PMC11123999

[CR201] Phillips B et al (2000) Epidemiology of restless legs symptoms in adults. Arch Intern Med 160(14):2137–2141 10904456 10.1001/archinte.160.14.2137

[CR202] Portas CM, Bjorvatn B, Ursin R (2000) Serotonin and the sleep/wake cycle: special emphasis on microdialysis studies. Prog Neurobiol 60(1):13–35 10622375 10.1016/s0301-0082(98)00097-5

[CR203] Qu W-M et al (2012) Honokiol promotes non-rapid eye movement sleep via the benzodiazepine site of the GABAA receptor in mice. Br J Pharmacol 167(3):587–598 22537192 10.1111/j.1476-5381.2012.02010.x PMC3449263

[CR204] Rabbani M, Sajjadi SE, Vaezi A (2015) Evaluation of anxiolytic and sedative effect of essential oil and hydroalcoholic extract of Ocimum basilicum L. and chemical composition of its essential oil. Res Pharm Sci 10(6):535–543 26779273 PMC4698864

[CR205] Raj A et al (2020) 1214 higher bedroom temperature associated with poorer sleep: data from over 3.75 million nights. Sleep 43:A464

[CR206] Ramakrishnan K (2007) Treatment options for insomnia. S Afr Fam Pract 49(8):34–41

[CR207] Randerath W et al (2022) Current and novel treatment options for obstructive sleep apnoea. ERJ Open Research 8(2):00126–02022 35769417 10.1183/23120541.00126-2022 PMC9234427

[CR208] Ranjbar-Slamloo Y, Fazlali Z (2019) Dopamine and noradrenaline in the brain; overlapping or dissociate functions? Front Mol Neurosci 12:334 32038164 10.3389/fnmol.2019.00334 PMC6986277

[CR209] Redeker NS et al (2019) Workplace interventions to promote sleep health and an alert, healthy workforce. J Clin Sleep Med. 15(4):649–657 30952228 10.5664/jcsm.7734 PMC6457507

[CR210] Redeker NS et al (2020) Effects of cognitive behavioral therapy for insomnia on sleep, symptoms, stress, and autonomic function among patients with heart failure. Behav Sleep Med. 18(2):190–202. 10.1080/15402002.2018.1546709 10.1080/15402002.2018.1546709 PMC6529289 30461315

[CR211] Reichert CF, Deboer T, Landolt HP (2022) Adenosine, caffeine, and sleep-wake regulation: state of the science and perspectives. J Sleep Res 31(4):e13597 35575450 10.1111/jsr.13597 PMC9541543

[CR212] Ren X-J et al (2020) Sedative and hypnotic effects and transcriptome analysis of polygala tenuifolia in aged insomnia rats. Chin J Integr Med 26(6):434–441 32240473 10.1007/s11655-020-3087-6

[CR213] Riemann D et al (2017) European guideline for the diagnosis and treatment of insomnia. J Sleep Res 26(6):675–700 28875581 10.1111/jsr.12594

[CR214] Riemann D et al (2022) Insomnia disorder: State of the science and challenges for the future. J Sleep Res 31(4):e13604 35460140 10.1111/jsr.13604

[CR215] Riemann D et al (2023) The European Insomnia Guideline: an update on the diagnosis and treatment of insomnia 2023. J Sleep Res 32(6):e14035 38016484 10.1111/jsr.14035

[CR216] Romero-Corral A et al (2010) Interactions between obesity and obstructive sleep apnea: implications for treatment. Chest 137(3):711–719 20202954 10.1378/chest.09-0360 PMC3021364

[CR217] Roth T (2007a) Insomnia: definition, prevalence, etiology, and consequences. J Clin Sleep Med 3(5 suppl):S7–S10 17824495 PMC1978319

[CR218] Rusch HL et al (2019) The effect of mindfulness meditation on sleep quality: a systematic review and meta-analysis of randomized controlled trials. Ann N Y Acad Sci 1445(1):5–16 30575050 10.1111/nyas.13996 PMC6557693

[CR219] Salehi B et al (2019) Melatonin in medicinal and food plants: occurrence, bioavailability, and health potential for humans. Cells 8(7):681 31284489 10.3390/cells8070681 PMC6678868

[CR220] Sánchez-Ortuño MM, Bélanger L, Ivers H, LeBlanc M, Morin CM (2009) The use of natural products for sleep: a common practice? Sleep Med 10(9):982–7 19427262 10.1016/j.sleep.2008.10.009

[CR221] Santos FA, Rao VSN (2000) Antiinflammatory and antinociceptive effects of 1,8-cineole a terpenoid oxide present in many plant essential oils. Phytother Res 14(4):240–244 10861965 10.1002/1099-1573(200006)14:4<240::aid-ptr573>3.0.co;2-x

[CR222] Sateia MJ (2014) International classification of sleep disorders-third edition: highlights and modifications. Chest 146(5):1387–1394 25367475 10.1378/chest.14-0970

[CR223] Sateia MJ et al (2017) Clinical practice guideline for the pharmacologic treatment of chronic insomnia in adults: an American Academy of Sleep Medicine clinical practice guideline. J Clin Sleep Med 13(02):307–349 27998379 10.5664/jcsm.6470 PMC5263087

[CR224] Sayyah M, Valizadeh J, Kamalinejad M (2002) Anticonvulsant activity of the leaf essential oil of Laurus nobilis against pentylenetetrazole- and maximal electroshock-induced seizures. Phytomedicine 9(3):212–216 12046861 10.1078/0944-7113-00113

[CR225] Scammell TE, Winrow CJ (2011) Orexin receptors: pharmacology and therapeutic opportunities. Annu Rev Pharmacol Toxicol 51:243–266 21034217 10.1146/annurev-pharmtox-010510-100528 PMC3058259

[CR226] Schiller H, Forster A, Vonhoff C, Hegger M, Biller A, Winterhoff H (2006) Sedating effects of Humulus lupulus L extracts. Phytomedicine 13(8):535–41 16860977 10.1016/j.phymed.2006.05.010

[CR227] Sepdanius E et al (2023) Relationship between physical activity, stress and sleep quality and emotional intelligence. Int J Hum Mov Sports Sci 11(1):224–232

[CR228] Shah R et al (2023) Mild sleep restriction increases endothelial oxidative stress in female persons. Sci Rep 13(1):15360 37717072 10.1038/s41598-023-42758-y PMC10505226

[CR229] Shan L et al (2022) Reduced numbers of corticotropin-releasing hormone neurons in narcolepsy type 1. Ann Neurol 91(2):282–288 34981555 10.1002/ana.26300 PMC9306683

[CR230] Shechter A et al (2020) Interventions to reduce short-wavelength (“blue”) light exposure at night and their effects on sleep: a systematic review and meta-analysis. Sleep Adv 1(1):zpaa002 37192881 10.1093/sleepadvances/zpaa002 PMC10127364

[CR231] Sheth S et al (2014) Adenosine receptors: expression, function and regulation. Int J Mol Sci 15(2):2024–2052 24477263 10.3390/ijms15022024 PMC3958836

[CR232] Shi YF, Yu YQ (2013) The roles of glutamate in sleep and wakefulness. Zhejiang Da Xue Xue Bao Yi Xue Ban 42(5):583–590 24167143

[CR233] Shi Y et al (2014) Herbal insomnia medications that target GABAergic systems: a review of the psychopharmacological evidence. Curr Neuropharmacol 12(3):289–302 24851093 10.2174/1570159X11666131227001243 PMC4023459

[CR234] Shirazi M et al (2021) The effectiveness of Melissa officinalis L. versus citalopram on quality of life of menopausal women with sleep disorder: a randomized double-blind clinical trial. Rev Bras Ginecol Obstet 43(2):126–130 33465795 10.1055/s-0040-1721857 PMC10183928

[CR235] Silber MH et al (2002) The epidemiology of narcolepsy in Olmsted County, Minnesota: a population-based study. Sleep 25(2):197–202 11902429 10.1093/sleep/25.2.197

[CR236] Silber MH et al (2021) The management of restless legs syndrome: an updated algorithm. Mayo Clin Proc 96(7):1921–1937 34218864 10.1016/j.mayocp.2020.12.026

[CR237] Simon KC et al (2022) Progressive muscle relaxation increases slow-wave sleep during a daytime nap. J Sleep Res 31(5):e13574 35355351 10.1111/jsr.13574 PMC9786620

[CR238] Smagula SF et al (2016) Risk factors for sleep disturbances in older adults: evidence from prospective studies. Sleep Med Rev 25:21–30 26140867 10.1016/j.smrv.2015.01.003 PMC4506260

[CR239] Smart TG, Stephenson FA (2019) A half century of gamma-aminobutyric acid. Brain Neurosci Adv 3:2398212819858249 32166183 10.1177/2398212819858249 PMC7058221

[CR240] Smith PC, Mong JA (2019) Neuroendocrine control of sleep. Curr Top Behav Neurosci 43:353–378 31396895 10.1007/7854_2019_107 PMC6986346

[CR241] Smolensky MH et al (2011) Sleep disorders, medical conditions, and road accident risk. Accid Anal Prev 43(2):533–548 21130215 10.1016/j.aap.2009.12.004

[CR242] Soltanpour A et al (2019) Effects of Melissa officinalis on anxiety and sleep quality in patients undergoing coronary artery bypass surgery: a double-blind randomized placebo controlled trial. Eur J Integr Med 28:27–32

[CR243] Soong C et al (2021) Advise non-pharmacological therapy as first line treatment for chronic insomnia. BMJ 372:n680 33757960 10.1136/bmj.n680

[CR244] Spadola CE et al (2019) Evening intake of alcohol, caffeine, and nicotine: night-to-night associations with sleep duration and continuity among African Americans in the Jackson Heart Sleep Study. Sleep 42(11):zsz136 31386152 10.1093/sleep/zsz136 PMC6802565

[CR245] Spalka J et al (2020) Morning headache as an obstructive sleep apnea-related symptom among sleep clinic patients-a cross-section analysis. Brain Sci 10(1):57 31963788 10.3390/brainsci10010057 PMC7016602

[CR246] Squires RF et al (1999) Honokiol and magnolol increase the number of [3H]muscimol binding sites three-fold in rat forebrain membranes in vitro using a filtration assay, by Allosterically Increasing the Affinities of Low-Affinity Sites. Neurochem Res 24(12):1593–1602 10591411 10.1023/a:1021116502548

[CR247] Strickland SR (2022) Sleep disorders. InnovAiT 16(1):27–33

[CR248] Strik H et al (2021) Why do our cancer patients sleep so badly? Sleep disorders in cancer patients: a frequent symptom with multiple causes. Oncol Res Treat 44(9):469–475 34350870 10.1159/000518108

[CR249] Strøm-Tejsen P et al (2016) The effects of increased bedroom air temperature on sleep and next-day mental performance. The 14th International Conference of Indoor Air Quality and ClimateAt: Ghent, Belgium, 640

[CR250] Sweetman A et al (2021) Effect of depression, anxiety, and stress symptoms on response to cognitive behavioral therapy for insomnia in patients with comorbid insomnia and sleep apnea: a randomized controlled trial. J Clin Sleep Med 17(3):545–554 33118927 10.5664/jcsm.8944 PMC7927315

[CR251] Taavoni S, Nazem Ekbatani N, Haghani H (2013) Valerian/lemon balm use for sleep disorders during menopause. Complement Ther Clin Pract 19(4):193–196 24199972 10.1016/j.ctcp.2013.07.002

[CR252] Taherzadeh Z et al (2020) Evaluation of sedative effects of an intranasal dosage form containing saffron, lettuce seeds and sweet violet in primary chronic insomnia: a randomized, double-dummy, double-blind placebo controlled clinical trial. J Ethnopharmacol 262:113116 32736046 10.1016/j.jep.2020.113116

[CR253] Tähkämö L, Partonen T, Pesonen A-K (2019) Systematic review of light exposure impact on human circadian rhythm. Chronobiol Int 36(2):151–170 30311830 10.1080/07420528.2018.1527773

[CR254] Taximaimaiti R, Luo X, Wang XP (2021) Pharmacological and non-pharmacological treatments of sleep disorders in Parkinson’s disease. Curr Neuropharmacol 19(12):2233–2249 33998990 10.2174/1570159X19666210517115706 PMC9185775

[CR255] Thakkar MM (2011) Histamine in the regulation of wakefulness. Sleep Med Rev 15(1):65–74 20851648 10.1016/j.smrv.2010.06.004 PMC3016451

[CR256] Thakkar MM, Winston S, McCarley RW (2003) A1 receptor and adenosinergic homeostatic regulation of sleep-wakefulness: effects of antisense to the A1 receptor in the cholinergic basal forebrain. J Neurosci 23(10):4278–4287 12764116 10.1523/JNEUROSCI.23-10-04278.2003 PMC2002519

[CR257] Thomas JM et al (2020) Circadian rhythm phase shifts caused by timed exercise vary with chronotype. JCI Insight 5(3):e134270. 10.1172/jci.insight.134270 10.1172/jci.insight.134270 PMC7098792 31895695

[CR258] Thorpy MJ, Bogan RK (2020) Update on the pharmacologic management of narcolepsy: mechanisms of action and clinical implications. Sleep Med 68:97–109 32032921 10.1016/j.sleep.2019.09.001

[CR259] Thorpy MJ, Siegel JM, Dauvilliers Y (2024) REM sleep in narcolepsy. Sleep Med Rev 77:101976 39186901 10.1016/j.smrv.2024.101976

[CR260] Tóth-Mészáros A et al (2023) The effect of adaptogenic plants on stress: a systematic review and meta-analysis. J Funct Foods 108:105695

[CR261] Toussaint L et al (2021) Effectiveness of progressive muscle relaxation, deep breathing, and guided imagery in promoting psychological and physiological states of relaxation. Evid Based Complement Alternat Med 2021(1):5924040 34306146 10.1155/2021/5924040 PMC8272667

[CR262] Turmel D et al (2022) Tailored individual Yoga practice improves sleep quality, fatigue, anxiety, and depression in chronic insomnia disorder. BMC Psychiatry 22(1):267 35421962 10.1186/s12888-022-03936-w PMC9012014

[CR263] Uchida K et al (2024) Effect of matcha green tea on cognitive functions and sleep quality in older adults with cognitive decline: a randomized controlled study over 12 months. PLoS ONE 19(8):e0309287 39213264 10.1371/journal.pone.0309287 PMC11364242

[CR264] Uehleke B et al (2012) Phase II trial on the effects of Silexan in patients with neurasthenia, post-traumatic stress disorder or somatization disorder. Phytomedicine 19(8–9):665–671 22475718 10.1016/j.phymed.2012.02.020

[CR265] Um MY et al (2019) Rice bran extract supplement improves sleep efficiency and sleep onset in adults with sleep disturbance: a randomized, double-blind, placebo-controlled, polysomnographic study. Sci Rep 9(1):12339 31451704 10.1038/s41598-019-48743-8 PMC6710429

[CR266] Umigai N, Takeda R, Mori A (2018) Effect of crocetin on quality of sleep: a randomized, double-blind, placebo-controlled, crossover study. Complement Ther Med 41:47–51 30477864 10.1016/j.ctim.2018.09.003

[CR267] US Preventive Services Task Force et al (2022) Screening for obstructive sleep apnea in adults: US preventive services task force recommendation statement. JAMA 328(19):1945–1950 36378202 10.1001/jama.2022.20304

[CR268] Van Cauter E, Plat L (1996) Physiology of growth hormone secretion during sleep. J Pediatr 128(5 Pt 2):S32–S37 8627466 10.1016/s0022-3476(96)70008-2

[CR269] Vanfleteren LE et al (2020) Multimorbidity in COPD, does sleep matter? Eur J Intern Med 73:7–15 31980328 10.1016/j.ejim.2019.12.032

[CR270] Varadharasu S, Das N (2024) Sleep hygiene efficacy on quality of sleep and mental ability among insomniac patients. J Family Med Prim Care 13(10):4693–4698 39629415 10.4103/jfmpc.jfmpc_48_24 PMC11610801

[CR271] Vgontzas AN et al (1997) Rapid eye movement sleep correlates with the overall activities of the hypothalamic-pituitary-adrenal axis and sympathetic system in healthy humans. J Clin Endocrinol Metab 82(10):3278–3280 9329353 10.1210/jcem.82.10.4307

[CR272] Vgontzas AN et al (2001) Chronic insomnia is associated with nyctohemeral activation of the hypothalamic-pituitary-adrenal axis: clinical implications. J Clin Endocrinol Metab 86(8):3787–3794 11502812 10.1210/jcem.86.8.7778

[CR273] Vgontzas A, Pavlovic J, Bertisch S (2023) Sleep symptoms and disorders in episodic migraine: assessment and management. Curr Pain Headache Rep 27(10):511–520 37665530 10.1007/s11916-023-01160-z

[CR274] Viola H et al (1995) Apigenin, a component of Matricaria recutita flowers, is a central benzodiazepine receptors-ligand with anxiolytic effects. Planta Med 61(03):213–216 7617761 10.1055/s-2006-958058

[CR275] Vyazovskiy VV, Cirelli C, Tononi G (2011) Electrophysiological correlates of sleep homeostasis in freely behaving rats. Prog Brain Res 193:17–38 21854953 10.1016/B978-0-444-53839-0.00002-8 PMC3160719

[CR276] Walker WH et al (2020) Circadian Rhythm Disruption and Mental Health 10(1):28. 10.1038/s41398-020-0694-0 10.1038/s41398-020-0694-0 PMC7026420 32066704

[CR277] Wang F, Boros S (2021) The effect of daily walking exercise on sleep quality in healthy young adults. Sport Sci Health 17:393–401

[CR278] Wang X et al (2019) The effect of mind-body therapies on insomnia: a systematic review and meta-analysis. Evid Based Complement Alternat Med 2019(1):9359807 30894878 10.1155/2019/9359807 PMC6393899

[CR279] Wang M et al (2020) Schisandrin B exerts hypnotic effects in PCPA-treated rats by increasing hypothalamic 5-HT and γ-aminobutyric acid levels. Exp Ther Med 20(6):1–1 33093880 10.3892/etm.2020.9271 PMC7571383

[CR280] Wang Q et al (2021) The role of sleep disorders in cardiovascular diseases: culprit or accomplice? Life Sci 283:119851 34324916 10.1016/j.lfs.2021.119851

[CR281] Weinert D, Gubin D (2022) The impact of physical activity on the circadian system: benefits for health, performance and wellbeing. Appl Sci 12(18):9220

[CR282] Welsh DK, Takahashi JS, Kay SA (2010) Suprachiasmatic nucleus: cell autonomy and network properties. Annu Rev Physiol 72:551–577 20148688 10.1146/annurev-physiol-021909-135919 PMC3758475

[CR283] Wilson D et al (2022) The effectiveness of a combined healthy eating, physical activity, and sleep hygiene lifestyle intervention on health and fitness of overweight airline pilots: a controlled trial. Nutrients 14(9):1988 35565955 10.3390/nu14091988 PMC9100076

[CR284] Wilson D et al (2023) Healthy nutrition, physical activity, and sleep hygiene to promote cardiometabolic health of airline pilots: a narrative review. J Lifestyle Med 13(1):1 37250274 10.15280/jlm.2023.13.1.1 PMC10210965

[CR285] Winkelman JW et al (2016) Practice guideline summary: treatment of restless legs syndrome in adults. Neurology 87(24):2585–2593 27856776 10.1212/WNL.0000000000003388 PMC5206998

[CR286] Wirth MD et al (2020) Changes in dietary inflammatory potential predict changes in sleep quality metrics, but not sleep duration. Sleep 43(11):zsaa093 32406919 10.1093/sleep/zsaa093 PMC7658634

[CR287] Wisden W, Yu X, Franks NP (2019) GABA receptors and the pharmacology of sleep. Handb Exp Pharmacol 253:279–304 28993837 10.1007/164_2017_56

[CR288] Woelk H, Schläfke S (2010) A multi-center, double-blind, randomised study of the Lavender oil preparation Silexan in comparison to Lorazepam for generalized anxiety disorder. Phytomedicine 17(2):94–99 19962288 10.1016/j.phymed.2009.10.006

[CR289] Xia TJ et al (2023) Melatonin-related dysfunction in chronic restraint stress triggers sleep disorders in mice. Front Pharmacol 14:1210393 37408758 10.3389/fphar.2023.1210393 PMC10318904

[CR290] Xiong ZY et al (2024) Efficacy of Chinese medicine treatment based on syndrome differentiation for primary insomnia: a randomized placebo controlled triple-blinded trial. Chin J Integr Med 30(10):867–876 38753273 10.1007/s11655-024-3661-4

[CR291] Yan M et al (2015a) Sedative and hypnotic effects of the extracts from Nelumbo nucifera leaves. Food Sci 36:168–171

[CR292] Yan M-Z et al (2015b) Lotus leaf alkaloid extract displays sedative–hypnotic and anxiolytic effects through GABAA receptor. J Agric Food Chem 63(42):9277–9285 26448283 10.1021/acs.jafc.5b04141

[CR293] Yeom JW, Cho CH (2024) Herbal and natural supplements for improving sleep: a literature review. Psychiatry Investig 21(8):810–821 39086164 10.30773/pi.2024.0121 PMC11321869

[CR294] Yi P-L et al (2007) The involvement of serotonin receptors in suanzaorentang-induced sleep alteration. J Biomed Sci 14:829–840 17657585 10.1007/s11373-007-9197-8

[CR295] Yildirim D et al (2025) The efficacy of lavender oil on fatigue and sleep quality in patients with hematological malignancy receiving chemotherapy: a single-blind randomized controlled trial. Support Care Cancer 33(2):79 39775962 10.1007/s00520-024-09143-5 PMC11711766

[CR296] Yin D et al (2019) Glutamate activates the histaminergic tuberomammillary nucleus and increases wakefulness in rats. Neuroscience 413:86–98 31202706 10.1016/j.neuroscience.2019.05.032

[CR297] Yoo DY et al (2011) Effects of Melissa officinalis L. (Lemon Balm) extract on neurogenesis associated with serum corticosterone and GABA in the mouse dentate gyrus. Neurochem Res 36(2):250–257 21076869 10.1007/s11064-010-0312-2

[CR298] Yoo J et al (2023) Daily reactivity to stress and sleep disturbances: unique risk factors for insomnia. Sleep 46(2):zsac256 36301838 10.1093/sleep/zsac256 PMC9905776

[CR299] Zanoli P, Avallone R, Baraldi M (2000) Behavioral characterisation of the flavonoids apigenin and chrysin. Fitoterapia 71:S117–S123 10930722 10.1016/s0367-326x(00)00186-6

[CR300] Zanoli P, Rivasi M, Zavatti M, Brusiani F, Baraldi M (2005) New insight in the neuropharmacological activity of Humulus lupulus L. J Ethnopharmacol 102(1):102–6 16046089 10.1016/j.jep.2005.05.040

[CR301] Zare Elmi HK et al (2021) Efficacy of valerian extract on sleep quality after coronary artery bypass graft surgery: a triple-blind randomized controlled trial. Chin J Integr Med 27(1):7–15 33420602 10.1007/s11655-020-2727-1

[CR302] Zeng L-N et al (2020) Gender difference in the prevalence of insomnia: a meta-analysis of observational studies. Front Psychiatry 11:577429 33329116 10.3389/fpsyt.2020.577429 PMC7714764

[CR303] Zhang C et al (2014) Pharmacological evaluation of sedative and hypnotic effects of schizandrin through the modification of pentobarbital-induced sleep behaviors in mice. Eur J Pharmacol 744:157–163 25446916 10.1016/j.ejphar.2014.09.012

[CR304] Zhang J et al (2022) The role of intracerebral dopamine D1 and D2 receptors in sleep-wake cycles and general anesthesia. Ibrain 8(1):48–54 37786416 10.1002/ibra.12024 PMC10528804

[CR305] Zhang Y et al (2022) Association of magnesium intake with sleep duration and sleep quality: findings from the CARDIA study. Sleep 45(4):zsab276 34883514 10.1093/sleep/zsab276 PMC8996025

[CR306] Zhao D et al (2019) Melatonin synthesis and function: evolutionary history in animals and plants. Front Endocrinol (Lausanne) 10:249 31057485 10.3389/fendo.2019.00249 PMC6481276

[CR307] Zhou Y et al (2022) Benefits of different combinations of aerobic and resistance exercise for improving plasma glucose and lipid metabolism and sleep quality among elderly patients with metabolic syndrome: a randomized controlled trial. Endocr J 69(7):819–830 35197411 10.1507/endocrj.EJ21-0589

[CR308] Zujko ME, Witkowska AM (2023) Dietary antioxidants and chronic diseases. Antioxidants (Basel) 12(2):362 36829921 10.3390/antiox12020362 PMC9952631

